# RECOVER identifies synergistic drug combinations *in vitro* through sequential model optimization

**DOI:** 10.1016/j.crmeth.2023.100599

**Published:** 2023-10-04

**Authors:** Paul Bertin, Jarrid Rector-Brooks, Deepak Sharma, Thomas Gaudelet, Andrew Anighoro, Torsten Gross, Francisco Martínez-Peña, Eileen L. Tang, M.S. Suraj, Cristian Regep, Jeremy B.R. Hayter, Maksym Korablyov, Nicholas Valiante, Almer van der Sloot, Mike Tyers, Charles E.S. Roberts, Michael M. Bronstein, Luke L. Lairson, Jake P. Taylor-King, Yoshua Bengio

**Affiliations:** 1 Mila, the Quebec AI Institute, Montreal, QC, Canada; 2 Relation Therapeutics, London, UK; 3 Department of Chemistry, The Scripps Research Institute, La Jolla, CA, USA; 4 Innovac Therapeutics, Inc., Cambridge, MA, USA; 5 IRIC, Institute for Research in Immunology and Cancer, Université de Montréal, Montreal, QC, Canada; 6 Department of Computer Science, University of Oxford, Oxford, UK; 7 Program in Molecular Medicine, Peter Gilgan Centre for Research and Learning, The Hospital for Sick Children, 686 Bay Street, Toronto, ON M5G 0A4, Canada

**Keywords:** machine learning, deep learning, active learning, sequential model optimization, drug combination, drug synergy, oncology, in vitro screening

## Abstract

For large libraries of small molecules, exhaustive combinatorial chemical screens become infeasible to perform when considering a range of disease models, assay conditions, and dose ranges. Deep learning models have achieved state-of-the-art results *in silico* for the prediction of synergy scores. However, databases of drug combinations are biased toward synergistic agents and results do not generalize out of distribution. During 5 rounds of experimentation, we employ sequential model optimization with a deep learning model to select drug combinations increasingly enriched for synergism and active against a cancer cell line—evaluating only ∼5% of the total search space. Moreover, we find that learned drug embeddings (using structural information) begin to reflect biological mechanisms. *In silico* benchmarking suggests search queries are ∼5–10× enriched for highly synergistic drug combinations by using sequential rounds of evaluation when compared with random selection or ∼3× when using a pretrained model.

## Highlights

• The RECOVER pipeline guides cell viability assays and selects drug combinations
• Increasing enrichment for synergism is achieved through five rounds of experiments
• *In silico* benchmarking suggests a ∼5–10× fold enrichment compared to random selection

## Motivation

Galvanized by the COVID-19 pandemic, we wanted to systematically identify efficacious drug combinations from the plethora of safe drugs that could hypothetically exhibit antiviral activity. The infeasibility of extensive combinatorial screens triggered the need for new methods that would require substantially less screening than an exhaustive evaluation. Outside of biology, there has been much interest in how areas of machine learning, including active learning and sequential model optimization, can be utilized to efficiently explore large spaces of possibilities through the intelligent acquisition and interpretation of data. Sequential model optimization has received much interest within biomedicine, with a focus on systems with well-described individual components, e.g., biomolecular design, chemical assays, etc. We wanted to apply a similar philosophy to quickly identify synergistic drug combinations to alter the phenotype of a cellular model system (cell viability as proof of concept), where the relationship between the chemical inputs and resulting phenotypic output is not well understood and is subject to experimental biases.

## Graphical abstract

**Figure:**
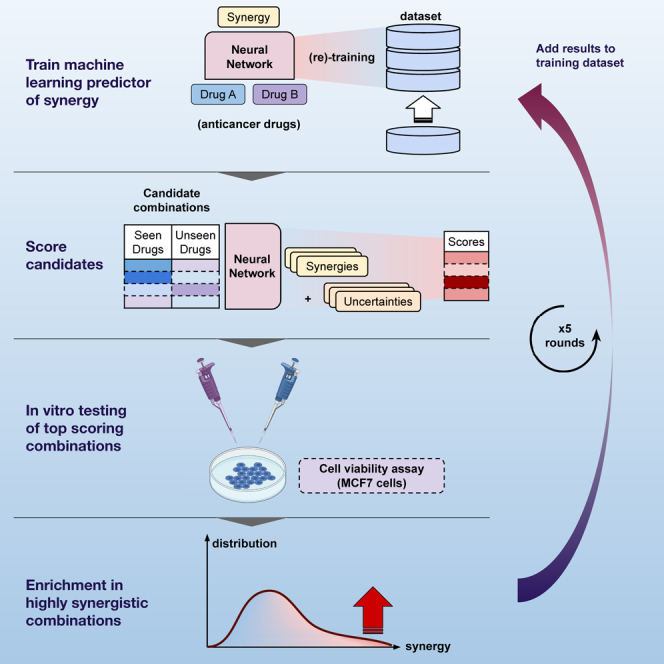

## Teaser

For large libraries of small molecules, exhaustive combinatorial screens become infeasible. Through five rounds of experimentation, Bertin et al. utilize a deep learning model to guide cell viability assays and select drug combinations, evaluating only ∼5% of the total search space while selecting drugs with increasing levels of synergism.

## Introduction

Drug combinations are an important therapeutic strategy for treating diseases that are subject to evolutionary dynamics, in particular cancers and infectious diseases.^1^^,^^2^ Conceptually, as tumors or pathogens are subject to change over time, they may develop resistance to a single agent^3^—motivating one to target multiple biological mechanisms simultaneously.^4^ Discovering synergistic drug combinations is a key step toward developing robust therapies, as they hold the potential for greater efficacy while reducing dose and hopefully limiting the likelihood of adverse effects. For example, in a drug repurposing scenario (i.e., uncovering new indications for known drugs), the ReFRAME library of ∼12,000 clinical-stage compounds^5^ leads to ∼72 million pairwise combinations; this does not appear tractable with standard high-throughput screening (HTS) technology—even at a single dose.^6^ Moreover, with patient-derived organoids (PDOs) being examined as a biomarker within personalized medicine clinical studies,^7^^,^^8^ the search space expands further to identify efficacious drug combinations specific to the mutation profile in question.

With the recent COVID-19 global health crisis, there has been the need for rapid drug repurposing that would allow for expedited and derisked clinical trials. Due to the complexity of selecting drug combinations and the minimal training data publicly available, studies have typically been limited toward monotherapy repurposing from a variety of angles—often involving artificial intelligence (AI) techniques to provide recommendations.^9^ The dearth of drug combination datasets is due to the large combinatorial space of possible experiments available—ultimately limiting the quality of drug synergy predictions. Moreover, databases of drug combinations are biased toward suspected synergistic agents, and thus making predictions outside the scope of the training dataset can be challenging.

The goal of this work is to discover synergistic drug combinations while only requiring minimal wet-lab experimentation. One cost-efficient tool at our disposal is sequential model optimization (SMO), whereby a machine learning (ML) model selects experiments (i.e., pairs of drugs) that it would like to be evaluated (in this case, for drug synergism). Both highly informative experiments (“exploration”) and experiments that double down on promising data-driven hypotheses (“exploitation”) can be selected.^10^ Between rounds of experimental evaluation, the model is iteratively adapted to new observations (via model training), which allows performance to gradually improve. This SMO process allows for queries that are more and more enriched with highly synergistic combinations, ultimately leading to reduced experimentation when compared to an exhaustive search.

There have now been a number of approaches for predicting the effects of and subsequently prioritizing drug combinations.^11^ Classic bioinformatics approaches have focused on using ML and network statistics over specified features of drugs (e.g., molecular fingerprints^12^), cell lines (e.g., transcriptomics, copy-number variations^13^), and interactome topology between biomolecules (e.g., protein-protein interactions, chemical-genetic interactions,^14^ or gene regulatory networks^15^). Initiatives such as the Dialogue on Reverse Engineering Assessment and Methods (DREAM) have led to a plethora of methods being benchmarked against one another in prospective challenges through the generation of novel datasets.^16^ Complex deep learning architectures, which have set state-of-the-art performance across a number of domains,^17^ have been used to predict both adverse drug-drug interactions^18^^,^^19^ and synergistic drug combinations.^20^^,^^21^^,^^22^ Sequential approaches, wherein several rounds of selection are performed, have also been explored in the context of drug combinations; for example, Kashif et al.^23^ have proposed a heuristic-based (as opposed to a model-based) exploration strategy.

We present a SMO platform that can guide wet-lab experiments: RECOVER, a deep learning regression model that predicts synergy using molecular fingerprints as inputs. To motivate the use of RECOVER, we demonstrate a real-world use case whereby one observes both: a ∼5–10× estimate for the enrichment of synergistic drugs identified using SMO when compared with selecting drug combinations at random and a ∼3× improvement when compared with selecting drugs in a single batch using a pretrained model. We then perform a retrospective validation to benchmark the performance of our model and understand its generalization abilities using the DrugComb database—largely pertaining to cancer cell line data.^24^ Thereafter, we evaluate our SMO pipeline *in silico*, which allows the model to select the most relevant data points to be labeled in order to discover the most promising combinations while reducing model uncertainty. Finally, we test RECOVER prospectively in an *in vitro* experimental setting, whereby we discover novel synergistic combinations active against a breast cancer model cell line, MCF7, which is also represented within our training dataset.

With an SMO platform available in conjunction with an appropriate *in vitro* assay, one has a powerful tool to rapidly respond to a future public health crisis. To encourage use by the scientific community, we detail a configuration that can be trained on a personal computer or laptop without requiring dedicated computational infrastructure. Remarkably, high predictive power is not a prerequisite for such an SMO system to be utilized effectively. In fact, as we are trying to identify pairs of drugs in prospective experiments that have more extreme synergy scores than those drug combinations evaluated within previous experiments (i.e., our training dataset), we cannot necessarily expect to have high predictive power. However, we achieve our ultimate goal: the identification of highly synergistic drugs—not building highly accurate ML models. This work forms a proof-of-concept demonstration of RECOVER—which should then motivate greater community adoption of the method and extensions thereof.

## Results

### RECOVER: SMO platform for rapid drug repurposing

**Figure 1:**
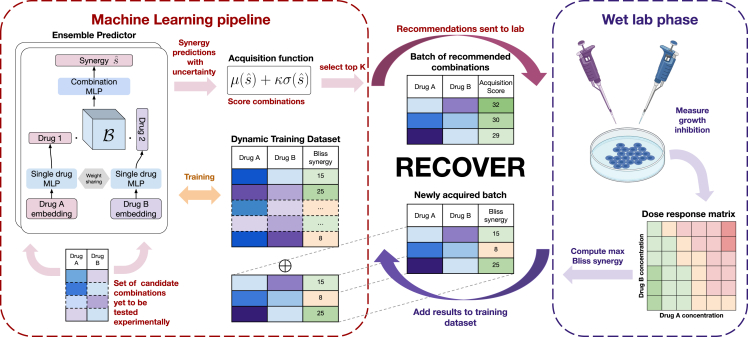
Overview of the RECOVER workflow integrating both a novel machine-learning pipeline and iterated wet-lab evaluation

RECOVER is an open-source SMO platform for the optimal suggestion of drug combinations (see Figure 1). Pairs of drug feature vectors are fed into a deep neural network, which is used for the prediction of synergy scores. These feature vectors include molecular fingerprints as well as a one-hot encoding identifying a drug. For a full description of the model, see method details and Figure S4A.

Our core focus is the prediction of pairwise drug combination synergy scores. While many mathematical descriptions of synergy have been proposed,^1^ in the following work, we utilize the Bliss synergy score due to its simplicity and numerical stability. In the context of cell viability, the Bliss independence model assumes that in the absence of synergistic effects, the expected fraction of viable cells after treatment with drugs $d_{1}$ and $d_{2}$ at doses $c_{1}$ and $c_{2}$, written $V\left(c_{1},c_{2}\right)$, is identical to the product of the fractions of viable cells when utilizing each drug independently, i.e., $V\left(c_{1}\right)V\left(c_{2}\right)$. We then define the Bliss synergy score as the difference between these quantities such that a fraction of surviving cells $V\left(c_{1},c_{2}\right)$ smaller than the expected proportion $V\left(c_{1}\right)V\left(c_{2}\right)$ leads to a large Bliss synergy score,

(Equation 1) $$s_{Bliss}\left(c_{1},c_{2}\right)=V\left(c_{1}\right)V\left(c_{2}\right)-V\left(c_{1},c_{2}\right)=I\left(c_{1},c_{2}\right)-I\left(c_{1}\right)-I\left(c_{2}\right)+I\left(c_{1}\right)I\left(c_{2}\right)\text{,}$$

where I(·)=1−V(·) is the experimentally measured growth inhibition induced by drug $d_{1}$, $d_{2}$, or both together at the associated doses. Given a dose-response matrix for the two drugs, a global synergy score can be obtained through a pooling strategy. In our case, we take the maximum value, i.e.,

(Equation 2) $${\hat{s}}_{Bliss}=\max_{c_{1},c_{2}}\;s_{Bliss}\left(c_{1},c_{2}\right)\;\text{.}$$

In many studies, the arithmetic mean is taken to calculate a global synergy score. Unfortunately, different laboratories use different dose intervals for each drug, and typically, each drug combination shows a synergistic effect at a specific dose-pair interval. Therefore, the arithmetic mean is highly sensitive to the chosen dose interval and is thus why we choose to prioritize a max-pooling strategy as in Equation 2. Unless explicitly stated otherwise, a synergy score refers to a global max-pooled Bliss score.

In addition to the prediction of synergy, RECOVER estimates the uncertainty associated with the underlying prediction. More precisely, for a given combination of drugs, RECOVER not only provides a point estimate of the synergy but estimates the distribution of possible synergy scores for each combination, which we refer to as the predictive distribution. We define the model uncertainty as the standard deviation of the predictive distribution.

An acquisition function is used to select the combinations that should be tested in subsequent experiments.^25^ This acquisition function is designed to balance between *exploration*, prioritizing combinations with high model uncertainty, whereby labeling said points should increase predictive accuracy in future experimental rounds; and *exploitation*, the selection of combinations believed to be synergistic with high confidence.

In summary, this SMO setting consists of generating recommendations of drug combinations that will be tested *in vitro* at regular intervals. At each step, RECOVER is trained on all the data acquired up to that point, and predictions are made for all combinations that could be hypothetically tested experimentally. The acquisition function is then used to provide recommendations for *in vitro* testing. The results of the experiments are then added to the training data for the next round of experiments, and the whole process repeats itself.

#### Task variations

We note that there are two separate but related frameworks in which RECOVER can be utilized.

In the preclinical framework, RECOVER can be used to recommend drug combinations expected to be effective within a single specified cell model system: the model is asked to provide synergy predictions from inputs $\left(d_{1},d_{2}\right)$ for drugs $d_{1}$ and $d_{2}$ and to subsequently provide recommendations in the same format. The preclinical framework is most relevant to early drug discovery; for example, one may wish to prioritize assets within a portfolio that synergize with an already approved drug. Naturally, we can apply RECOVER to any disease areas where *in vitro* cell models are routinely used in early drug discovery, e.g., collagen deposition (fibrosis), T cell activation (immunology), etc.

In an alternative setup, the personalized framework requires RECOVER to recommend drug combinations expected to be effective in one or more available model systems: the model is asked to provide predictions and subsequent recommendations of the form $\left(d_{1},d_{2},m\right)$ for drugs $d_{1}$, $d_{2}$, and model system *m*. The personalized framework is most relevant to novel personalized cancer treatment scenarios, wherein multiple patient-derived primary models are available and recommendations are sought to optimize the use of approved drugs in a highly translatable but low-throughput system.^26^^,^^27^

#### Illustration of SMO approach

To illustrate the benefits of the SMO approach, we perform a preliminary simulation to mimic a scientist with a limited experimental budget of 300 drug combinations to be tested—with the aim to find synergistic drug combinations. We assume that the experimentalist has access to a trained ML model, and we show the benefit of RECOVER within both frameworks. At a high level, we specify that there are two options: either to perform all 300 experiments in one go, or to perform experiments in 10 batches of 30.

We note that many ML papers focus on the personalized framework,^20^^,^^28^^,^^29^^,^^30^ i.e., recommendations are of the form $\left(d_{1},d_{2},m\right)$, so we demonstrate the benefit of SMO in this scenario first. All models are pretrained on the O’Neil drug combination study,^31^ and validation by the experimentalist is simulated through uncovering specific examples from the NCI-ALMANAC drug combination study^32^ restricted to all cell lines that are covered in both studies. In more detail, we test the following options: random, all 300 combinations are queried at random; DeepSynergy, the synergies of all combinations in ALMANAC are predicted using the DeepSynergy model with the top 300 predictions queried; RECOVER without SMO, the synergies of all combinations in ALMANAC are predicted using the RECOVER model with the top 300 predictions queried; RECOVER, 30 combinations are queried at random followed by an SMO using batches of 30; and DeepSynergy with SMO, which is the same SMO as before but using the DeepSynergy model.

**Figure 2:**
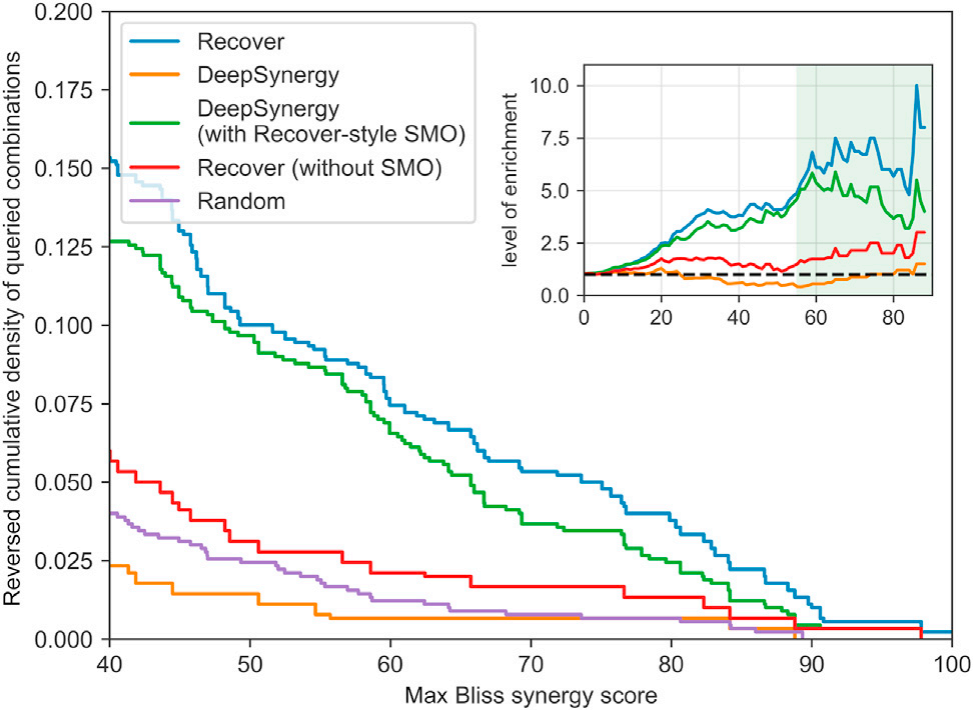
Simulations suggest that RECOVER can enrich for highly synergistic combinations given a limited budget Reversed cumulative density of queried combinations following different querying strategies. (Inset) Level of enrichment. Shaded area corresponds to synergies >54.9. Results are averaged over 3 seeds.

In Figure 2, we report the reversed cumulative density of the synergies of all 300 queried combinations (higher is better). We also report the level of enrichment defined as the ratio between the reversed cumulative density of a given strategy’s queries and the reversed cumulative density of random queries. We first observe that DeepSynergy^20^ performs worse than random, while RECOVER (without SMO) performs slightly above the level of randomness. Most importantly, the bulk of the performance gain comes from utilizing our SMO procedure. Finally, when RECOVER and DeepSynergy are compared head to head in the SMO setting, the RECOVER model outperforms the DeepSynergy model.

The threshold for “highly synergistic” is challenging to specify, but we note that a drug combination in clinical trials has a max Bliss synergy score of 54.9 (see discovery and rediscovery of novel synergistic drug combinations). On this basis, these experiments suggest that our approach can reduce by a factor of ∼5–10× the number of experiments needed to discover and validate highly synergistic drug combinations when compared with random selection or by a factor of >3× when using a pretrained model selecting all drug combinations at a single time point.

For completeness, we show in Figure S1A that we achieve a broadly similar level of enrichment when evaluating a preclinical framework task for three different cell lines. The experimental setup is exactly the same except that the search space is now restricted to a specific cell line within the NCI-ALMANAC study and recommendations are of the form $\left(d_{1},d_{2}\right)$. We note that tasks drawn from the preclinical framework are slightly more challenging than the tasks drawn from the personalized framework, as the model cannot evaluate the same drug pairs in new cell lines (which would likely lead to drug synergy), and so the performance is marginally lower.

#### Scope of RECOVER capabilities and experimental validation

Due to the operational complexities in prospectively evaluating performance in the personalized framework, we focus on the preclinical framework for experimental proof of concept and demonstration of the RECOVER system. In Figure S1H, we report key aspects of our prospective validation and how it compares with the ones performed in other published works. We note that other works focused on generalizing to a new cell line and/or combinations of drugs both seen during training. Our prospective validation focuses on testing the ability of RECOVER to generalize to combinations involving one drug seen during training and one unseen drug, which is a harder task. In addition, validation involves, for the first time, repeated experimentation via an integrated wet-lab/dry-lab system.

### Retrospective testing of RECOVER informs the design of future experiments

In preparation for prospective validation within the preclinical framework, we evaluate the performance of RECOVER *in silico* using previously published data. In order to understand the scope of scenarios to which RECOVER can be applied to, we benchmark RECOVER against baseline models and test our ability to generalize in several out-of-distribution tasks without incorporating SMO. Thereafter, we perform backtesting through simulating mock SMO experiments (see SMO development and evaluation in the method details, as well as in Figures S4D–S4F).

Due to the limited size of most individual drug combination studies reported in the literature, we focus on the NCI-ALMANAC viability screen^32^ summarized in Figure S1B. We refrain from combining multiple datasets because of the severe batch effects between studies; in Figure S1F, we show a scatterplot that demonstrates inconsistency between the O’Neil et al.^31^ series of drug combination experiments against their NCI-ALMANAC counterpart. We note that this may result from variation in the readouts of these experiments, mutations in cell lines, or differences in harvest times.

**Figure 3:**
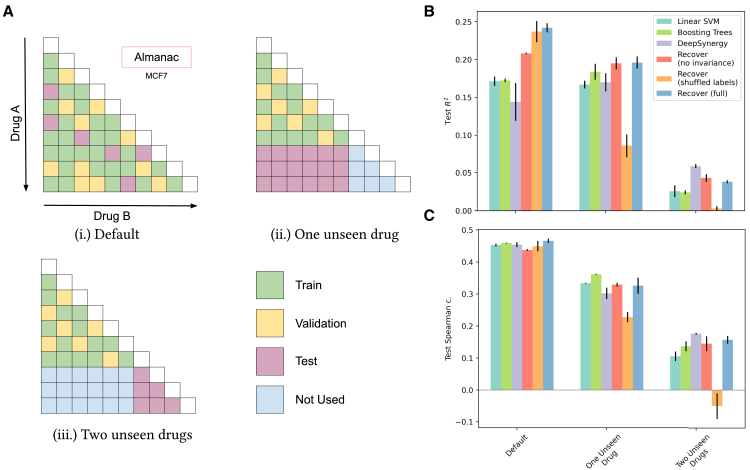
Retrospective testing demonstrates the ability of RECOVER **t**o generalize when at least one of the drugs has been seen during training but not beyond that (A) Overview of the different tasks on which RECOVER has been evaluated in preparation for the prospective evaluation within the preclinical framework. Each task corresponds to a different way to split the training, validation, and test sets and aims at evaluating a specific generalization ability of the model. (i.) Default. Combinations are split randomly into training/validation/test (70%/20%/10%). Only the MCF7 cell line is used. (ii.) One unseen drug. 30% of available drugs are excluded from the training and validation sets. The test set consists of combinations between a drug seen during training and an unseen drug. Combinations among seen drugs are split into training and validation (80%/20%). Only the MCF7 cell line is used. (iii.) Two unseen drugs. Similar to task (ii.), but the test set consists of combinations of two unseen drugs. (B and C) Performance of RECOVER and other models for the three different tasks. Standard deviation computed over 3 seeds.

We investigate whether RECOVER can generalize beyond the training (and validation) set in various ways: (Figure 3Ai.) what is the performance on test cases drawn from the same distribution as the training set? Can RECOVER generalize when (Figure 3Aii.) one of the drugs is unseen (during training) or (Figure 3Aiii.) when both of the drugs are unseen? These tasks are illustrated graphically in Figure 3A. For each task, we benchmark against several alternative models along with RECOVER, including a linear support vector machine (SVM), Boosting Trees, and DeepSynergy.^20^ In addition, we evaluate a version of RECOVER without the invariance module and another version for which the identities of the drugs (as well as cell lines) have been shuffled (see model development and evaluation in the method details for further information on models and hyperparameter optimization procedures). Through understanding the capability of RECOVER to generalize, we can design prospective experiments with a greater confidence of success.

In Figures 3B and 3C, we report the test performance metrics of RECOVER across each of the first three tasks. Examining performance within task (i.) in Figure 3A, test statistics appear modest; however, we demonstrate limits on achievable performance—resulting from experimental noise and non-uniformity of synergy scores (see Figure S2F). From task (i.) to task (iii.) in Figure 3A, we note a drastic drop in performance for all models, but this effect is alleviated if only one of the drugs has not been seen before (see task ii. in Figure 3A). We also investigate additional scenarios from the personalized framework, presented in Figure S2A, wherein we consider multiple cell lines, as well as training and test sets coming from different studies, and report performance in Figure S2B.

We note that our benchmarking justifies various aspects of our deep learning architecture: the RECOVER permutation invariance module can provide improvement in performance across some scenarios; moreover, RECOVER (shuffled labels) fails compared with other methods on task (ii.) in Figure 3A with one unseen drug and is at the level of randomness on task (iii.) in Figure 3A with two unseen drugs. In these cases, we demonstrate that drug structure is actually leveraged by the model in order to generalize (to some extent) to unseen drugs. However, RECOVER (shuffled labels) performs well compared with other models on the default task; thus, merely knowing the identity of the drugs is sufficient when both drugs have been seen in other combinations.

From the above results, we can recommend that any prospective experiments should require that one of the two drugs in the combination have been seen in some context before (see task iii. in Figure 3A). Due to the severe batch experiments between studies in the public domain, as shown in Figure S1F, models fail to generalize to data coming from a different study, as shown in Figure S2B (study transfer task). As such, should we want to utilize publicly available resources, we will have to incorporate such data intelligently. To this end, we investigated using transfer learning, wherein one trains a model on a large dataset (known as pretraining) and thereafter refines the model on a smaller dataset (known as fine-tuning)—typically with some aspect of the task or the data changed between the two instances. We show that this is possible and beneficial (compared to not leveraging existing data) in an SMO setting between the O’Neil et al.^31^ and NCI-ALMANAC studies (see Figure S4E). Remarkably, even with minimal correlation between studies, we are able to observe the benefits of transfer learning in this scenario. These findings suggest that we use transfer learning within prospective experiments.

### Prospective use of RECOVER enriches for selection of synergistic drug combinations

From the *in silico* results, we now test RECOVER prospectively using a cancer cell model, leveraging publicly available data for pretraining. Using the insights from retrospective testing of RECOVER informs the design of future experiments, the queriable space of drug combinations was designed to include drug pairs where only one compound was already seen by the model during pretraining—with a second compound not seen before. For details about the model used to generate recommendations, see recommendation generation in the method details. The MCF7 cell line was used to generate 6×6 dose-response matrices (see experimental protocol for details).

We perform multiple rounds of RECOVER-informed wet-lab experiments and observe sequential improvements in performance. The rounds of experiments are described as follows.

(1) Calibration. The initial round of experiments was performed to supplement publicly available data with 20 randomly selected unseen drug combinations. Furthermore, we confirmed the previous *in silico* result that we could not predict synergy scores (prior to transfer learning adaptation) through selecting 5 highly synergistic combinations selected by RECOVER. In addition, 5 more drug combinations were selected by a graph neural network (GNN) model in the style of Zitnik et al.^18^ that we did not develop further due to the computational overhead. It was also specified that each drug should appear in, at most, a single drug combination queried.
(2) Diversity. Thereafter, drug combinations are selected using model predictions in conjunction with the upper confidence bound (UCB) acquisition function. To ensure that we quickly observe all single drugs at least once (as we showed that the model cannot generalize well to combinations involving unseen drugs), we select our batch of experiments as follows. First, we rank combinations according to their acquisition function score. We then find the first combination that involves a drug that has not yet been used (or that is involved in one of the combinations from the current batch) and add it to the batch. We repeat this until we have 30 combinations in the batch.
(3) SMO search. RECOVER is now free to select any drug pairs of interest for testing, with the requirement that any single drug may be selected no more than 5 times (to avoid oversampling and depletion of chemical stock). Three such rounds have been performed in this manner.

**Figure 4:**
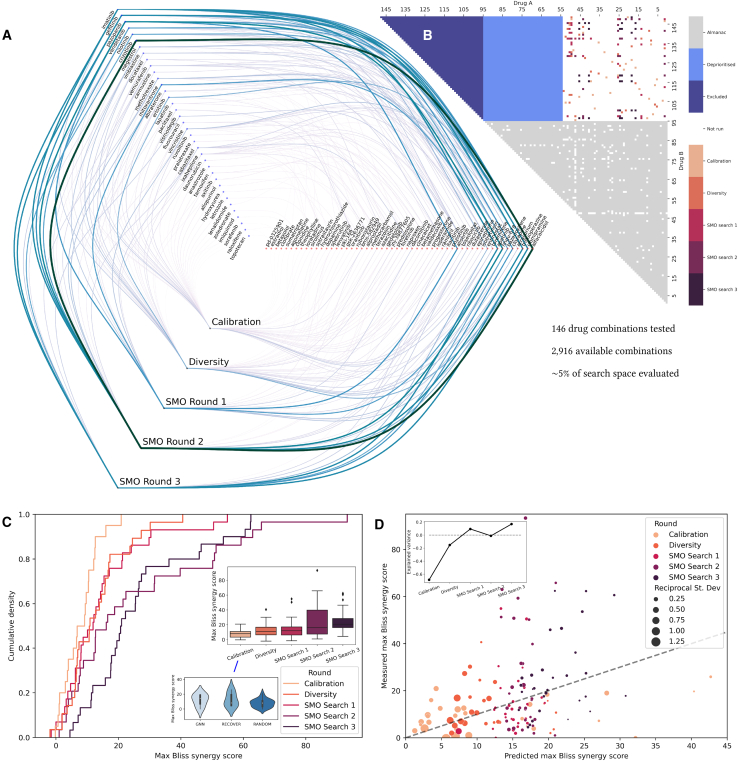
*In vitro* evaluation demonstrates the significant enrichment for highly synergistic combinations through prospective use of RECOVER (A) Network plot indicating which pairs of drugs were identified at each round; line color and width represent synergy. (B) Heatmap representing drug combinations used during pretraining (NCI-ALMANAC), in the five subsequent rounds of experiments, and combinations excluded from the analysis. Drug combinations that were not available for pretraining or were not selected for experiments are represented in white. (C) Cumulative density plot of max Bliss synergy score for each experimental round; (inset) boxplot representation and calibration round details. (D) Predicted versus actual plot for max Bliss synergy score. The dotted line corresponds to y=x. (Inset) The explained variance is plotted for each experimental round. See also Data S1 and Tables S1 and S2.

The search space was constructed as follows. The NCI-ALMANAC includes 95 unique drugs that were employed in combinations tested on the MCF7 cell line (see gray area in Figure 4B). We chose to deprioritize drugs without a well-characterized mechanism of action (MoA) to facilitate biological interpretation and validation of the results (see light blue area in Figure 4B). To achieve this, drugs in NCI-ALMANAC were annotated with known targets extracted from the ChEMBL drug mechanism table: 54 drugs matched with at least one known target were thus selected. An additional 54 drugs were selected by clustering drugs with known MoAs that are included in the DrugComb^24^ database but not in NCI-ALMANAC. Hence, a search space including a total of 2,916 drug combinations was obtained (see the white area in Figure 4B). In Figure 4A, we illustrate the pairs of drugs selected in each round of experiments.

We now evaluate both the synergy scores of the drug combinations selected and the underlying accuracy of the model. In Figure 4C, we plot the cumulative density function of each experimental round. We note that the mean of the max Bliss synergy scores significantly increases between the first and the third rounds (t test, p<0.05); this trend further continues by the fifth round (t test, $p<10^{-5}$). Moreover, the distribution starts developing a heavier tail toward high max Bliss synergy scores. This emergent heavy tail already appears significant when comparing the distribution in the first SMO search round to the background distribution of synergy scores in NCI-ALMANAC (Kolmogorov-Smirnov test, p<0.025). Finally, the highest max Bliss synergy score observed increases between rounds until the second SMO search round, whereby the behavior appears to have saturated. These results are focused on the max Bliss score, which RECOVER was specifically designed to optimize for; for completeness, we also report similar evaluations based on different aggregation strategies of the Bliss scores (see Figure S3A).

All combinations queried throughout the five rounds, and their corresponding synergy scores, are provided in Table S1. We notice that specific drugs tend to appear in several of the combinations recommended by RECOVER. Consistent with the literature, we observe that some compounds appear more often than others within synergistic combinations,^33^ a pattern that can also be observed within the NCI-ALMANAC study (see Figure S1C). However, this does not make the identification of synergistic combinations a trivial problem: even drugs that lead to the highest number of synergistic combinations are non-synergistic most of the time. No single drug within the NCI-ALMANAC study has a synergy score >40 more than 10% of the time (or 12% when considering only the MCF7 cell line data within the NCI-ALMANAC study; see Figure S1G). In comparison, our last two rounds of *in vitro* experiments yielded 20%–30% of combinations with a synergy >40 (see Figure 4C), while the model had only observed less than 5% of the search space.

In Figure 4D, we plot the predicted versus actual plot of the max Bliss synergy score. Here, the point size in the scatterplot is inversely proportional to the model uncertainty; therefore, we display confident predictions as large points, and vice versa. As expected, more confident predictions are closer to the y=x line. Less-confident predictions are associated with larger max Bliss synergy scores. Moreover, we systematically underestimate the measured max Bliss synergy score (more points far above y=x line); this intuitively makes sense, as we are trying to identify highly synergistic drug combinations that are not within our training dataset. Figure 4D (inset) displays the increase in (weighted) explained variance from one round to the next; weights are chosen to be the reciprocal of the model uncertainty. We find that, initially, the explained variance is negative, i.e., our model has no predictive power. However, as the experiments continue, a positive trend emerges such that we have a small amount of predictive power by the end of the experiments.

This increase in performance and in the synergy of queried combinations from one round to the next demonstrated in Figure 4C is expected and can be attributed to two factors. First, we needed to adapt the model to predict in a new experimental setting. From the study transfer task in Figure S2A, we know that this would otherwise be an impossible task and thus motivates the calibration round. After the calibration round, one expects that the systematic biases learned by the model during pretraining are minimized. At this point, the model is in a scenario akin to task (ii.) in Figure 3A. Second, we can improve performance further by enforcing that (almost) all drugs have been evaluated at some point, which subsequently motivated the diversity round. Thereafter, the model is free to optimize during the SMO rounds to the extent that it is able to, leveraging model predictions and model uncertainties. In fact, due to activity cliff effects,^34^ there are likely fundamental limits on quantifying the relationship between model uncertainty and model error; in Figures S4B and S4C, we perform a preliminary investigation of these relationships. From our prospective use of RECOVER, we not only discover highly synergistic drug combinations but also demonstrate that high predictive power is not strictly necessary to identify synergistic drug combinations.

### Discovery and rediscovery of novel synergistic drug combinations

In Data S1, we provide detailed information on our experimental results using the Combenefit package^35^ (including single-agent dose-response curves, combination dose-response surfaces, and synergy levels) for the 14 most synergistic drug combinations (from the ∼150 tested), with alfacalcidol and crizotinib achieving a max Bliss score above 90. Of note, we rapidly discover drug combinations with similar mechanisms and efficacy to those already in clinical trials. Namely, within the first SMO search round we found (1) alisertib and pazopanib and (2) flumatinib and mitoxantrone. The concentration intervals for the drugs used in both drug combinations that show synergy are consistent with therapeutically relevant plasma concentrations^36^^,^^37^ or as observed in *in vivo* animal experiments (flumatinib).^38^

Pazopanib inhibits angiogenesis through targeting a range of kinases including vascular endothelial growth factor receptor (VEGFR), platelet-derived growth factor receptor (PDGFR), c-KIT, and fibroblast growth factor receptors (FGFRs); in contrast, alisertib is a highly selective inhibitor of mitotic Aurora A kinase. Synergism between the two agents is hypothesized to be linked to the observation that mitosis-targeting agents also demonstrate antiangiogenic effects. In an independent study, the combination of alisertib and pazopanib has successfully completed phase 1b clinical trials for advanced solid tumors.^36^ The combination of flumatinib and mitoxantrone appears to be linked to a similar mechanism but does not seem to have been studied in the biomedical literature. While flumatinib is a tyrosine kinase inhibitor targeting Bcr-Abl, PDGFR, and c-KIT, mitoxantrone is a type II topoisomerase inhibitor.

**Figure 5:**
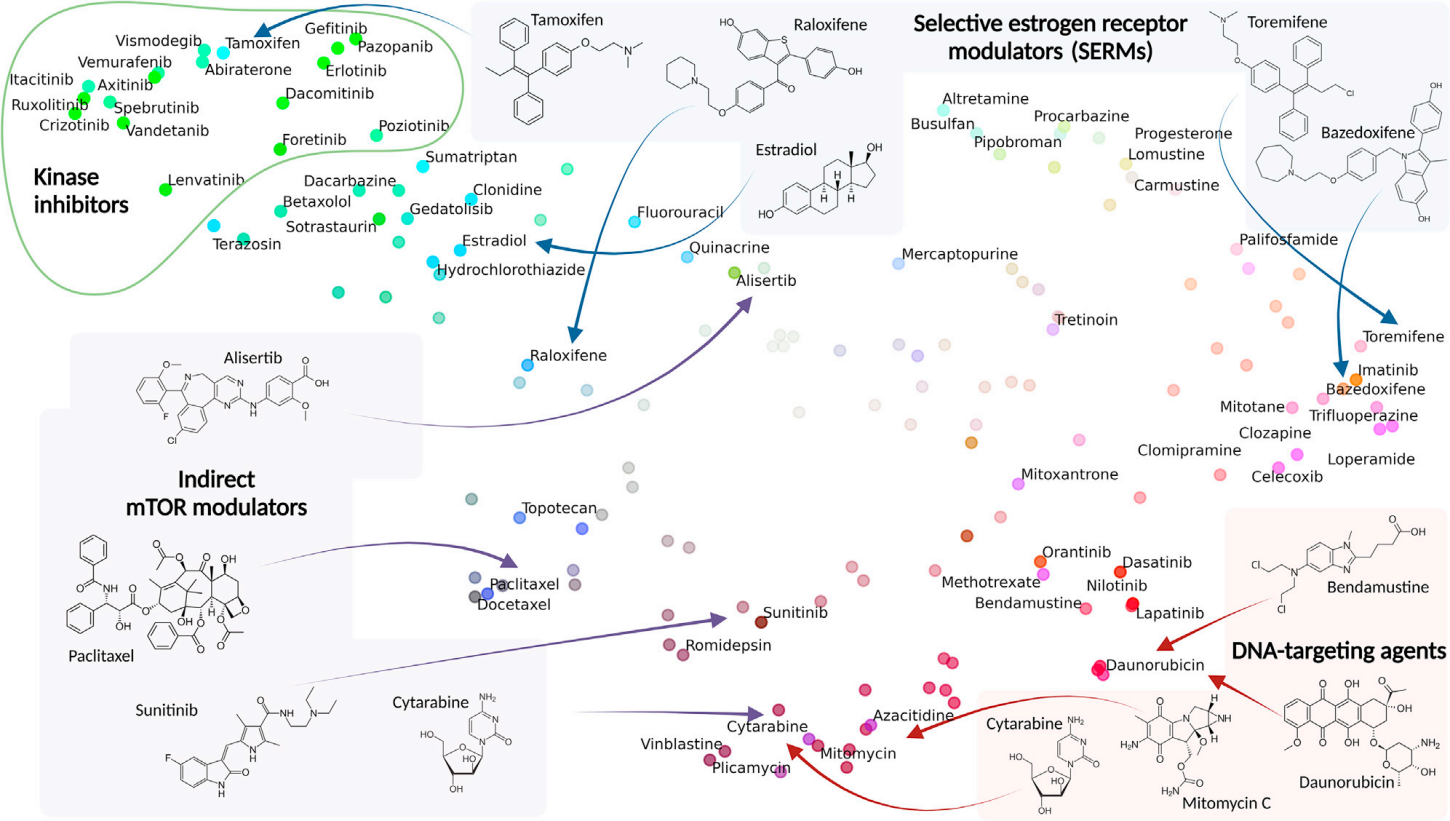
RECOVER tends to map molecules with common biological mechanisms closely together (reflected by the similar colors of nearby points), even when structures are dissimilar UMAP of RECOVER drug embeddings with the color scheme generated to indicate the known target profile of the drugs; drugs that have molecular targets in common will have similar colors. Drug embeddings are learned using information from drug structures and viability screen data only.

RECOVER drug embeddings capture both structural and biological information. To get a better insight into the drug embeddings learned by RECOVER, we report uniform manifold approximation and projection (UMAP) visualizations of the drug embeddings generated by the single-drug module in Figure 5. The color of each point is chosen by applying principal-component analysis (PCA) to the binary matrix of drug-targets and scaling the first 3 dimensions into an RGB triplet; high transparency indicates drugs with a PCA target profile close to the average PCA target profile (calculated over all drugs). In short, the position of the points indicates what RECOVER has learned about the drugs, and the color represents information known about drug mechanisms from other databases not used in the training procedure.

We note that the RECOVER model does not use information on drug targets; however, drugs with similar colors are located within similar areas of UMAP space. We also observe broad sensible patterns in UMAP space based on structure; for example, most kinase inhibitors (with the -*nib* suffix) appear in the top left hand of the UMAP. Moreover, drugs with similar mechanisms tend to be co-located; for example, see structurally diverse DNA-targeting agents in the bottom right of the UMAP. As a counterpoint, we observe that agents with either mixed agonist/antagonist profiles, including selective estrogen receptor modulators (SERMs), or targeting genes through indirect mechanisms, including mammalian target of rapamycin (mTOR), lead to less structured patterns in UMAP space. We believe that this is a highly novel observation and that it suggests that were this screen to be scaled to a larger library of small molecules, one may be able to group diverse structures into common biological mechanisms.

## Discussion

Drug combinations can achieve benefits unattainable by mono-therapies and are routinely investigated within clinical trials (e.g., PD-1/PD-L1 inhibitors combined with other agents^39^) and utilized within clinical practice (e.g., antiretroviral treatment of HIV where between 3 and 4 agents may be used^40^). To this end, we have presented the SMO toolbox RECOVER for drug combination identification. We have demonstrated its ability to generalize to combinations involving one unseen drug, and crucially, we have shown the benefit of repeated experimentation via an integrated wet-lab/dry-lab system. We showcase a general methodology, consisting of careful analysis of the properties of our ML pipeline—such as its out-of-distribution generalization capacities—to help us design key aspects of our prospective experiments, to eventually ensure a smooth and successful interaction between the SMO pipeline and the wet lab. Highly synergistic drug combinations have been identified, and the resulting learned embeddings appear to capture both structural and biological information. RECOVER can quickly (in our prospective experiments: <5% of the total search space evaluated) identify patterns in the drug-drug landscape of synergies, in order to provide recommendations significantly enriched for synergism and alleviate the need for exhaustive studies. We provide commentary on key aspects on our approach covering datasets, computational methodology, wet-lab techniques, and evaluation metrics.

We note the considerable difficulties of working with publicly available datasets with discrepancies in the data generation process. Inconsistent media between multiple labs, the presence of *de novo* mutations within immortalized *in vitro* cell models, and differences in experimental protocols limit ease of data integration between laboratories.^41^ In particular, systematic biases limit generalizability of model predictions to subsequent prospective experiments. Within oncology, protein-coding mutations may drive resistance to any one chemotherapeutic agent but also large-scale gene dosing changes from non-coding mutations,^42^ copy-number variations,^43^ and aneuploidy.^44^ These issues have been somewhat alleviated through careful choice of metric to optimize (e.g., max pooled Bliss synergy scores have reduced sensitivity to selected drug concentration ranges, compared to averaged scores) and only using publicly available data for pretraining (when compared with using these data for prediction without adaptation).

From a computational perspective, we experimented with a range of more complicated models. For example, we considered using GNNs to model biomolecular interactions,^45^ which have numerous benefits including greater biological interpretability and incorporation of prior knowledge, namely drug-target and protein-protein interactions. However, these models only resulted in marginal increases in performance while requiring substantially more computational resources. We believe that the limited diversity of the dataset and the simplicity of the task, a one-dimensional regression, did not allow these more advanced approaches to reach their full potential. Therefore, we prioritized a strategy that could be run quickly for rapid turnaround of recommendations for experimental testing.

When considering an SMO setting, we are required to collapse highly complex information into a single number to be optimized (i.e., a synergy score). While there is an opportunity to improve choices of metric (synergy scores may not reflect absolute cell viability), assay readouts that better characterize cell state (compared with cell viability) may provide a stronger starting point. In particular, an omics readout, through transcriptomics^46^ and/or single-cell profiling,^47^^,^^48^ and high content imaging^49^ provide a much higher-dimensional measurement of cell state. Furthermore, derived properties from these readouts may be more interpretable, e.g., pathway activation^50^ or extracellular signaling.^51^ Remarkably, even while only using cell viability as a readout, we achieved significant progress in identifying novel synergistic drug combinations.

Furthermore, the usual metrics for the evaluation and training of regression models may not reflect well the efficiency of models in iterative settings. This is due to the fact that, in our SMO setting, only the prediction of extreme values is important. This work provides an example of this effect: model performance on prospectively queried combinations was modest, but a substantial enrichment was achieved. Some metrics have been proposed to focus specifically on the prediction of extreme values.^52^ Developing training objectives that specifically aim at maximizing SMO performance will be the object of future work.

From the systematic screen by Jaak et al.,^33^ they conclude that synergy between drugs is rare and highly context dependent. RECOVER provides a means to identify such synergies while requiring substantially less screening than an exhaustive evaluation; thus, we expect that RECOVER and similar such systems may have a role to play when addressing diverse application areas such as personalized cancer treatment and novel emergent infectious disease such as the COVID-19 pandemic.

### Limitations of the study

In addition to the points mentioned above, a few restrictions were necessary in the name of feasibility concerning the validation experiments. In particular, only one cell model was used for validation, and the exhaustive evaluation of every possible drug combination was not performed. With regard to the downstream analysis, while we investigated the relationship between drugs and their mechanisms of action, many such mechanisms are not fully elucidated. Finally, our investigation into the relationship between the structural similarity of drug pairs, their synergy, the associated model error, and model uncertainty is preliminary in nature.

## STAR★Methods

### Key resources table

**Table:** 

REAGENT or RESOURCE	SOURCE	IDENTIFIER
**Deposited data**		

Reservoir package (parsed data, data acquisition scripts and parsing pipelines)	This paper	https://github.com/RECOVERcoalition/Reservoir

**Experimental models: Cell lines**		

MCF7 cells	ATCC	HTB-22

**Software and algorithms**		

Recover package	This paper	https://doi.org/10.5281/zenodo.8156097; https://github.com/RECOVERcoalition/Recover

**Chemicals, peptides, and recombinant proteins**		

5-Azacytidine	MedChem Express	HY-10586
5-Fluorouracil	MedChem Express	HY-90006
6-Thioguanine	MedChem Express	HY-13765
Abiraterone acetate	MedChem Express	HY-75054
Adavosertib (MK-1775)	Selleck	S1525
Agomelatine	MedChem Express	HY-17038
Alfacalcidol	Selleck	S1468
Alfacalcidol	MedChem Express	HY-10003
Alisertib	MedChem Express	HY-10971
Allopurinol	MedChem Express	HY-B0219
Alprenolol hydrochloride	MedChem Express	HY-B1517A
Altretamine	MedChem Express	HY-B0181
Anastrozole	MedChem Express	HY-14274
Apatinib	Selleck	S5248
Apilimod	Selleck	S6414
Atracurium (besylate)	MedChem Express	HY-B0292A
Axitinib	MedChem Express	HY-10065
Azathioprine	MedChem Express	HY-B0256
Baricitinib (INCB028050)	Selleck	S2851
Bazedoxifene (acetate)	MedChem Express	HY-A0036
Betamethasone dipropionate	MedChem Express	HY-13571
Betaxolol (hydrochloride)	MedChem Express	HY-B0381A
BMS-582949 (hydrochloride)	MedChem Express	HY-14305A
Bortezomib	MedChem Express	HY-10227
Butoconazole nitrate	MedChem Express	HY-B0293
Cabazitaxel	MedChem Express	HY-15459
Calcitriol (RO215535)	Selleck	S1466
Camostat Mesilate	Selleck	S2874
Candesartan	MedChem Express	HY-B0205
Captopril	MedChem Express	HY-B0368
Carbamazepine	MedChem Express	HY-B0246
Carmustine	MedChem Express	HY-13585
Celecoxib	MedChem Express	HY-14398
Chloramphenicol	MedChem Express	HY-B0239
Chloroquine	MedChem Express	HY-17589A
Cinacalcet (hydrochloride)	MedChem Express	HY-70037A
Clodronic acid disodium salt	MedChem Express	HY-B0657A
Clofazimine	Selleck	S4107
Clofibrate	MedChem Express	HY-B0287
Clomipramine hydrochloride	MedChem Express	HY-B0457
Clonidine	MedChem Express	HY-12721
Clozapine	MedChem Express	HY-14539
Crizotinib	MedChem Express	HY-50878
Cyclosporin A	Selleck	S2286
Cytarabine	MedChem Express	HY-13605
Dacomitinib	MedChem Express	HY-13272
Danoprevir (ITMN-191)	Selleck	S1183
Dasatinib	Selleck	S1021
Daunorubicin (RP 13057) HCl	Selleck	S3035
Decamethonium Bromide	MedChem Express	HY-B0570
Diclofenac Sodium	MedChem Express	HY-15037
Diphenhydramine hydrochloride	MedChem Express	HY-B0303A
Disulfiram	MedChem Express	HY-B0240
Docetaxel (Trihydrate)	MedChem Express	HY-B0011A
Dopamine HCl	Selleck	S2529
Doxycycline	Selleck	S5159
Dutasteride	MedChem Express	HY-13613
Ebastine	Selleck	S4262
EIDD-1931	Selleck	S0833
Entinostat	MedChem Express	HY-12163
Epinephrine HCl	Selleck	S3061
Erlotinib (Hydrochloride)	MedChem Express	HY-12008
Estradiol	MedChem Express	HY-B0141
Estrone	MedChem Express	HY-B0234
Exemestane	MedChem Express	HY-13632
Felbamate	MedChem Express	HY-B0184
Fenbufen	MedChem Express	HY-B1138
Fenoprofen Calcium hydrate	MedChem Express	HY-B0288B
Fludarabine	MedChem Express	HY-B0069
Flumatinib (mesylate)	MedChem Express	HY-13905
Fluoxetine HCl	Selleck	S1333
Flupentixol dihydrochloride	MedChem Express	HY-15856B
Foretinib	MedChem Express	HY-10338
Fulvestrant	MedChem Express	HY-13636
Gedatolisib (PKI-587)	Selleck	S2628
Gefitinib	MedChem Express	HY-50895
GSK2636771	MedChem Express	HY-15245
Guanfacine hydrochloride	MedChem Express	HY-17416
Haloperidol	MedChem Express	HY-14538
Hexestrol	MedChem Express	HY-B1662
Hydrochlorothiazide	MedChem Express	HY-B0252
Hydroxychloroquine sulfate	MedChem Express	HY-B1370
Hydroxyurea	MedChem Express	HY-B0313
Ibuprofen	MedChem Express	HY-78131
Imatinib	MedChem Express	HY-15463
Imiquimod (hydrochloride)	MedChem Express	HY-B0180A
Indomethacin	MedChem Express	HY-14397
Irinotecan	MedChem Express	HY-16562
Irsogladine	MedChem Express	HY-B0327
Isotretinoin	MedChem Express	HY-15127
Itacitinib	MedChem Express	HY-16997
Ivermectin	Selleck	S1351
Ixabepilone	MedChem Express	HY-10222
JNJ-38877605	MedChem Express	HY-50683
Ketoprofen	MedChem Express	HY-B0227
KU-60019	Selleck	S1570
Lapatinib	MedChem Express	HY-50898
Lenalidomide	MedChem Express	HY-A0003
Lenvatinib (E7080)	Selleck	S1164
Lenvatinib (E7080) Mesylate	Selleck	S5240
Letrozole	MedChem Express	HY-14248
Levofloxacin	MedChem Express	HY-B0330
Lidocaine	MedChem Express	HY-B0185
Loperamide (hydrochloride)	MedChem Express	HY-B0418A
Loratadine	MedChem Express	HY-17043
Maprotiline hydrochloride	MedChem Express	HY-B0444
Mefenamic acid	MedChem Express	HY-B0574
Megestrol acetate	MedChem Express	HY-13676
Mephenesin	Selleck	S5032
Methocarbamol	MedChem Express	HY-B0262
Methotrexate	MedChem Express	HY-14519
Methyldopa hydrate	MedChem Express	HY-B0225B
Mitotane	MedChem Express	HY-13690
Mitoxantrone (dihydrochloride)	MedChem Express	HY-13502A
MK-2206 2HCl	Selleck	S1078
Nafamostat mesylate	MedChem Express	HY-B0190A
Naproxen	MedChem Express	HY-15030
Nilotinib (monohydrochloride monohydrate)	MedChem Express	HY-10159A
Orantinib	MedChem Express	HY-10517
Paclitaxel	MedChem Express	HY-B0015
Pamidronate disodium pentahydrate	MedChem Express	HY-B0730
Pazopanib	Selleck	S3012
Pazopanib	MedChem Express	HY-10208
PD0325901	MedChem Express	HY-10254
Pemetrexed	MedChem Express	HY-10820
Pentamidine isethionate	MedChem Express	HY-B0537B
Phenylbutazone	MedChem Express	HY-B0230
Phenytoin	MedChem Express	HY-B0448
PKI-166	MedChem Express	HY-117155
Poziotinib	MedChem Express	HY-15730
Pralatrexate	MedChem Express	HY-10446
Prednisolone	MedChem Express	HY-17463
Primaquine Diphosphate	MedChem Express	HY-12651
Procarbazine Hydrochloride	MedChem Express	HY-13733
Progesterone	MedChem Express	HY-N0437
Prostaglandin E1	MedChem Express	HY-B0131
Raloxifene (hydrochloride)	MedChem Express	HY-13738A
Ramipril	MedChem Express	HY-B0279
Rapamycin	MedChem Express	HY-10219
Remdesivir (GS-5734)	Selleck	S8932
Retinoic acid	MedChem Express	HY-14649
Ribavirin	Selleck	S2504
Ribociclib	MedChem Express	HY-15777
Risedronate Sodium	Selleck	S1428
Ritonavir	Selleck	S1185
Romidepsin	MedChem Express	HY-15149
Ruboxistaurin (LY333531) HCl	Selleck	S7663
Ruxolitinib Phosphate	Selleck	S5243
S-(+)-Ketoprofen	MedChem Express	HY-B2137
Saracatinib (AZD0530)	Selleck	S1006
Selumetinib (AZD6244)	Selleck	S1008
Silmitasertib	MedChem Express	HY-50855
Sorafenib	MedChem Express	HY-10201
Sotrastaurin	MedChem Express	HY-10343
Spebrutinib	MedChem Express	HY-18012
Stigmasterol	MedChem Express	HY-N0131
Succinylcholine Chloride Dihydrate	Selleck	S4121
Sumatriptan (succinate)	MedChem Express	HY-B0121
Sunitinib	MedChem Express	HY-10255A
Tamoxifen	Selleck	S1238
Teniposide	MedChem Express	HY-13761
Terazosin (hydrochloride dihydrate)	MedChem Express	HY-B0371A
Thalidomide	MedChem Express	HY-14658
Theophylline	MedChem Express	HY-B0809
Topotecan (Hydrochloride)	MedChem Express	HY-13768A
Toremifene (citrate)	MedChem Express	HY-B0005
Tosedostat	MedChem Express	HY-14807
Trifluoperazine	Selleck	S5856
Trifluoperazine 2HCl	Selleck	S3201
Valdecoxib	MedChem Express	HY-15762
Vandetanib	MedChem Express	HY-10260
Vemurafenib	MedChem Express	HY-12057
Vinblastine (sulfate)	MedChem Express	HY-13780
Vincristine sulfate	Selleck	S1241
Vismodegib	MedChem Express	HY-10440
Vismodegib (GDC-0449)	Selleck	S1082
Vorinostat	MedChem Express	HY-10221
Zoledronic acid (monohydrate)	MedChem Express	HY-13777A

**Other**		

DMEM	Thermo Fisher Scientific	Cat #10569010
FBS	Thermo Fisher Scientific	Cat #10438026
Antibiotic-Antimycotic	Thermo Fisher Scientific	Cat #15240062
MycoAlert Mycoplasma Detection Kit	Lonza	Cat #LT07-218

### Resource availability

#### Lead contact

Further information and requests for resources should be directed to and will be fulfilled by the lead contact, Jake P. Taylor-King (jake@relationrx.com).

#### Materials availability

All viability data and synergy scores of prospectively validated drug combinations are provided in the form of two spreadsheets available as supplementary materials.

### Experimental model and study Participant details

MCF7 cells (female epithelial cells) were obtained from ATCC and maintained in DMEM (Thermo Fisher Scientific) supplemented with 10% FBS (Thermo Fisher Scientific) and Antibiotic-Antimycotic (Thermo Fisher Scientific) at 37°C in 5% ${\text{CO}}_{2}$ in a humidified incubator. Before the screens, the cell lines were passaged twice after thawing. Cultures were confirmed to be free of mycoplasma infection using the MycoAlert Mycoplasma Detection Kit (Lonza). Identifiers are listed in the Key resources table.

### Method details

#### Model description

We frame the problem of pairwise drug synergy prediction as a regression task $\left(\left\{d_{1},d_{2}\right\},\hat{s}\right)$: given a pair of drugs $d_{1},d_{2}$, we aim to predict their (pooled) level of synergy, $\hat{s}$. Our proposed architecture is an end-to-end deep learning model trained with a *mean square error* (MSE) criterion.

Our model can be decomposed into two modules. First, a *single drug* module, *E*, which produces representations (or embeddings) for the drugs based on their chemical structure information. The embeddings from a pair of drugs are used as input to the *combination* module *P*, which directly estimates the synergy score; see Figure S4A.

Further, uncertainty estimation methods are used in order to estimate the *predictive distribution* of synergies $p\left(\hat{s}|\left\{d_{1},d_{2}\right\}\right)$ for each drug pair $\left\{d_{1},d_{2}\right\}$, as opposed to a point estimate. The predictive distributions of drug pairs are given as input to an acquisition function in order to decide which combinations should be tested *in vitro*, balancing between combinations that are informative, i.e., that can reduce the generalization error of the model later on, and combinations that are likely to be synergistic.

##### Single drug module

Let $X_{D}\in R^{n_{D}\times l_{D}}$ denote the matrix of drug features, where $n_{D}$ is the number of drugs in D and $l_{D}$ corresponds to the number of raw features that describe each drug. Drug features used in this work include molecular fingerprints^12^ and one-hot encoding of the drugs.

The single drug module can be written as a function $E:D\to R^{k_{D}}$ where $k_{D}$ corresponds to the dimension of the output vector representation (or embedding) of each drug. Our single drug module is a simple multi-layer perceptron (MLP) that takes raw features of drugs as input and outputs an updated vector representation; this MLP can be conditioned on cell line (described below).

##### Combination module

Given a set of drugs D, the combination module corresponds to a function $P:\left(\begin{array}{l} D\\ 2 \end{array}\right)\mapsto R$ that maps a pair of drugs to their Bliss synergy score. We remark first that *P* should be agnostic to the order of the two drugs. Hence, the first operation of *P* correspond to a permutation invariant function – such as element-wise sum, mean, or max operations – applied to the two vector representations corresponding to each drug. In this work, we use a bilinear operation defined by a tensor $B\in R^{k_{D}\times k_{D}\times k}$, where *k* is a hyperparameter corresponding to the dimension of the vector representation of a drug combination. To ensure permutation invariance, we enforce that every slice across the third dimension (denoted as $B_{i}$), is a symmetric matrix. Note that we do not enforce $B_{i}$ to be positive definite, hence $B_{i}$ does not necessarily define a scalar product. The output of this permutation invariant function is fed to an MLP that outputs the predicted synergy for the pair of drugs; as before, the MLP can be conditioned on cell lines.

##### Cell line conditioning

As a drug effect is context dependent, the synergy of a combination of two drugs can be different in experiments using different cell lines. To account for the cell line in our model we condition upon it using FiLM.^53^ In essence, the FiLM approach learns an affine transformation of the activation of each neuron in the MLP.

We denote the matrix of cell line features by $X_{C}\in R^{n_{C}\times l_{C}}$, with C the set of cell lines, $n_{C}$ corresponding to the number of cell lines in C and $l_{C}$ giving the number of raw features for each cell line.

The feature representation of the cell line is either based on a one-hot encoding, or on information about mutations and basal level of gene expression. The former approach relies on having data for each cell line in the training set and cannot generalise to new cell lines; the latter approach makes use of features that represent cell lines.

#### Searching the space of drug combinations

##### Uncertainty estimation

Estimating the uncertainty of the predictions is a key step toward providing reliable recommendations as well as driving the exploration with SMO. For this purpose, we use a common uncertainty estimation method: deep ensembles.^54^ Given an ensemble of models which differ only in the initialization of the parameters, the predictions of the different models are considered as samples from the *predictive* distribution. In this work, we define uncertainty as the standard deviation of the *predictive* distribution, and can be estimated from the standard deviation between the predictions of the different members of the ensemble. Unless specified otherwise, we use a deep ensemble of size 5 as the uncertainty estimation method in our *in silico* experiments, and of size 36 for generation of wet lab recommendations.

Note that for completeness, we investigated other methods for uncertainty quantification in some of the *in silico* experiments, including direct estimation of the standard deviation of the predictive distribution — in a similar fashion to Direct Epistemic Uncertainty Prediction (DEUP),^55^ see SMO development and evaluation for details.

##### Sequential model optimization

Sequential model optimization (SMO) aims at discovering an input $x^{⋆}\in X$ maximizing an objective function *S*:

(Equation 3) $$x^{⋆}\in \underset{x\in X}{\arg\;\max}S\left(x\right)\text{.}$$

The SMO approach consists in tackling this problem by iteratively querying the objective function *S* in order to find a maximizer $x^{⋆}$ in a minimal number of steps. At each step *t*, the dataset is augmented such that $D_{t}$ contains all the inputs that have already been acquired at time *t*. The dataset $D_{t}$ is then used to find the next query $x^{\left(t+1\right)}$. In the context of drug combinations, *x* corresponds to a pair of drugs, and the objective function *S* corresponds to the synergy score.

SMO has been prospectively applied to: optimize the production of proteins in cell free systems^56^; determine gene functions in yeast^57^; enhance the production of fine chemicals in *Escherichia coli*^58^; and to identify inhibitors of *Mycobacterium tuberculosis* growth.^59^

In what follows, *f* refers to an estimator of the objective function *S*. One may notice that several properties of the potential queries $x^{\left(t\right)}$ should be taken into account. One would like to find an $x^{\left(t\right)}$ that would be informative to acquire (i.e., the uncertainty at $x^{\left(t\right)}$ is high) in order to obtain a reliable estimator of the objective function early on. On the other hand, one would like to find an $x^{\left(t\right)}$ that is a *good guess* in the sense that $f\left(x^{\left(t\right)}\right)$ is close to the expected maximum $\max_{x\in X}f\left(x\right)$. Looking for queries which are informative is referred to as *exploration* while looking for queries which are expected to maximize the objective function is called *exploitation*.

The key challenge of SMO is to balance between exploration and exploitation. This is typically achieved by designing an acquisition function (or strategy) α which defines a score on the space of inputs X and takes into account both the expected f(x) and an estimate of the uncertainty at *x*. The input which maximizes the score α is chosen as the next query. An overview of the SMO approach is presented in Algorithm 1.

Algorithm 1 **Sequential model optimization**

**Input:** Initial data $D_{0}$, objective function estimator *f*

**for** t∈{1,2,…} **do**

 Select new $x^{\left(t+1\right)}$ by optimizing an acquisition function α

 
 
 
 
 
 
 
 
$x^{\left(t+1\right)}=\underset{x}{\arg\;\max}\alpha \left(x;f\right)$

 Query objective function *S* to obtain $y^{t+1}$

 Augment data $D_{t+1}=D_{t}\cup \left\{\left(x^{t+1},y^{t+1}\right)\right\}$

 Update estimator *f*

**end for**.

In what follows, we assume that we have access to an estimate of the mean of the predictive distribution, $\hat{\mu }\left(x\right)$, as well as an estimate of the uncertainty $\hat{\sigma }\left(x\right)$. The key acquisition functions considered are detailed below.

###### Brute-force

α(x) corresponds to random noise, and therefore the drug combinations are selected at random.

###### Greedy

$\alpha \left(x\right)=\hat{\mu }\left(x\right)$. This acquisition function corresponds to pure exploitation whereby we select drug combinations with the highest predicted synergy.

###### Pure exploration

$\alpha \left(x\right)=\hat{\sigma }\left(x\right)$. This acquisition function corresponds to pure exploration. The strategy aims at labeling the most informative examples in order to reduce model uncertainty as fast as possible, and corresponds to the traditional strategy in *Active Learning*.

###### Upper confidence bound (UCB)

$\alpha \left(x\right)=\mu \left(x\right)+\kappa \hat{\sigma }\left(x\right)$. This strategy balances between exploration and exploitation. κ∈R is a hyperparameter that is typically positive. Higher values of κ give more importance to exploration.

Unless specified otherwise, *in silico* experiments involving SMO were performed using UCB with κ=1.

For all experiments, the model is reinitialized and trained from scratch on all (visible) data after each query. Whilst not designed for optimal computational efficiency, this procedure ensures that the model is not overfitting on examples that have been acquired early on.

#### Recommendation generation

In order to generate the recommendations for *in vitro* experiments, we trained 3 models using 3 different seeds on the NCI-ALMANAC study, restricting ourselves to samples from the MCF7 cell line. We refer to these 3 models as pretrained.

Afterward, we *fine-tune* using prospectively generated data. More precisely, the weights of one of the pretrained models were loaded, and some additional training was performed on prospectively generated data only, using early stopping. This fine-tuning process was repeated with 12 different seeds for each pretrained model. The end result being that we obtain an ensemble of 36 fine-tuned models in total.

This ensemble was used to generate predictions $\left(\hat{\mu },\hat{\sigma }\right)$ for all candidate combinations. We then use UCB with κ=1 to obtain a score according to which all candidates were ranked.

#### Dataset processing

Below, the major data types included in the RESERVOIR data repository are briefly described.

##### Drugs

Data on drugs and biologically active compounds has been extracted from Chembl,^60^ pre-processed and indexed with unique identifiers. A translation engine has been provided such that a compound can be translated to a unique identifier using generic or brand drug names, SMILES strings and Pubchem^61^ CIDs.

##### Cell line features

Additionally, RESERVOIR retrieved cell line features from the Cancer Dependency Map.^62^ These include genetic mutations, base level gene expression and metadata.^24^

##### Drug combinations

Literature drug combination data was extracted from DrugComb version 1.5.^24^ Quality control was applied on the experiments in DrugComb. Only blocks (i.e., combination matrices) complying to the following criteria were selected: (a.) filter out erroneous blocks that show very low variance, specifically inhibition standard deviation ≤ 0.05, (b.) filter out small blocks less than 3 × 3 dimensions, (c.) filter out blocks with extreme inhibition values, such that 5% < [mean pooled growth inhibition] < 95%.

The dataset used for model pretraining and *in silico* experiments consists of 4463 data points relative to experiments on MCF7 cell line expressed as max Bliss which were reported in the Almanac study. These data correspond to 4271 unique drug combinations made up by 95 unique drugs.

The prediction set for experiment selection was built by taking 54 out of the 95 Almanac drugs for which a mechanism of action (MoA) was annotated in ChEMBL 25.^60^ An additional 54 drugs were obtained by clustering 719 drugs with known MoA that are included in DrugComb but are not part of Almanac. Clustering was performed with the *k*-medoids algorithm as implemented in scikit-learn 0.24.2^63^ (n_clusters = 54, metric = Tanimoto similarity, init = k-medoids++), drugs were encoded by Morgan fingerprints with radius 2 and 1024 bits calculated with RDKit.^64^ A representative compound for each cluster was obtained by taking the cluster centroid.

Three of the centroid drugs were replaced due to lack of availability from commercial vendors or due to poor reported solubility. Replacements for each of the three drugs were selected by taking the nearest analogue (evaluated by Tanimoto similarity) in the same cluster. 54 Almanac and 54 non-Almanac compounds thus selected were used to build a set of 2916 binary combinations made up by one Almanac and one non-Almanac compound.

#### Experimental protocol

Results for all prospective experiments are included in Table S2.

Compounds were plated as a 6 × 6 dose-response combination matrix in natural 384 well plates (Greiner), in a serial 1:3 dilutions of each agent (5 concentrations) and only DMSO as the lowest concentration. We used a combination plate layout where six compound pairs could be accommodated on one 384 well plate. A set of control wells with DMSO was included on all plates as negative control. To ensure reproducibility and comparability with the subsequent combination studies, the IC50 of Doxorubicin was used as reference in a 6-point dose response format in each plate as positive (total killing) control. In addition, alfacalcidol and erlotinib were evaluated in multiple rounds (and excluded from our analysis) to ensure consistency in max Bliss synergy scores.

Cells were seeded in white 384-well plates (Greiner) at 1000 cells/well in 50 μL of media using a multidrop dispenser and allowed to attach for 2 h. Compounds from pre-plated matrix plates were transferred to each well using a 100 nL head affixed to an Agilent Bravo automated liquid handling platform, and plates were incubated at 37°C in 5% CO2 for an additional 72 h. To measure the cell viability, CellTiter-Glo reagent (diluted 1:6 in water, Promega) was dispensed into the wells (30 μL), incubated for 3 min, and luminescence was read on a Envision plate reader (PerkinElmer). Final DMSO concentration in assay wells was 0.2%. The assay was performed with 3 biological replicates.

The compound-specific concentration ranges were selected based on their published activities. In brief, dose ranges for each drug were selected based on the highest quality evidence available pertaining to the drug in question. Highest to lowest quality evidence sources were as follows.

1. IC50/range of concentrations available from DrugComb^24^ available in correct cell line (MCF7).
2. IC50/range of concentrations available for same cell line from any literature source.
3. IC50/range of concentrations available from DrugComb for a range of cell lines. Mean/median calculated and manually curated to assess any obvious difference for related cell lines.
4. IC50/range of concentrations available from the literature for any cell line.
5. Looking at prescribed dose in man and comparing with IC50s for similar prescribed doses for compounds in DrugComb.

For experimental ease, standard sets of concentrations were used for different sets of drugs, and IC50 ranges were rounded appropriately. For some compounds there were also limits for solubility which resulted in adjustment. All compounds were pre-diluted in DMSO to a stock concentration that varied from 10 to 50 mM, depending on the final concentration range required for each compound.

#### Combenefit preprocessing

We report some detailed results regarding the 14 top scoring prospectively tested combinations in Data S1. These plots were generated using the Combenefit package.^35^

In each case, we report the single agent dose-response curves (with EC 95/EC50 values estimated via Combenefit), as well as the combination dose-response data, both in matrix format and visualized as a surface. We also report the synergy levels in matrix format and projected on the combination dose-response surface, according to three different synergy models: HSA, Bliss and Loewe. Statistical significance (one sample *t* test) is computed and reported elementwise (∗ $p<5x10^{-2}$, ∗∗ $p<10^{-3}$, ∗∗∗ $p<10^{-4}$; the number of replicates (N) is shown on the top left corner of the matrix display). Note that the colormap only accounts for statistically significant values ($p<5\times 10^{-2}$).

Before analysis with Combenefit, all viability data has been normalized as follows.

• Define 0% as the viability percentage for the highest concentration of doxorubicin (DOX) per replicate.
• Define 100% as the viability percentage for the 0,0 μM control per combination.
• Cap and scale viability values between 0 and 100.

For reference, we also report single agent dose-response viability curves before normalization in Figure S3B. Finally, we report the average and maximum synergy of the 14 combinations, for each of the three models (HSA, Bliss and Loewe) in Figure S3C. Some drugs are over-represented and appear in several of these 14 top scoring combinations. The over-representation of some drugs among highly synergistic combinations is a well known phenomenon,^33^ and illustrated in Figure S1C.

Due to differences in processing compared to our standard pipeline, we notice small differences between values reported in the rest of the paper and values reported by Combenefit. For instance, the max Bliss score for Crizotinib & Alfacalcidol previously reported (93) is slightly lower than the estimate from Combenefit (99.99). This difference comes from the fact that we used the actual single agent responses for our independence model (as defined in Equation 1), while Combenefit relies on fitted Hill functions.

In a few combinations, we noted a high variability between replicates. If this variability is the result of a technical issue, it may lead to biases in our estimation of the synergy of these combinations. See for instance Imatinib & Clozapine (single agent response of Imatinib at 10μM), and Nilotinib & Clomipramine (single agent response of Clomipramine). Still, in both cases, synergy appears significant according to Combenefit’s one sample t test (p value $<10^{-4}$ and p value $<10^{-2}$ respectively for the two combinations mentioned above).

Some combinations, such as Flumatinib & Mitoxantrone, showcase a region of antagonism that is not accounted for by the max Bliss synergy score. The choice of max Bliss synergy as our metric is motivated by its reduced sensitivity to selected concentration ranges, which was necessary when using pretrained deep learning models. In future work, we can optimize the synergy score along with other metrics (e.g., antagonism, PKPD properties) and the RECOVER SMO approach could be used in the same manner but with a different objective. This work serves as a proof of concept, and more advanced implementations can be the object of future research.

#### Model development & evaluation, excluding SMO

We investigated various aspects of the performance of RECOVER for the prediction of Bliss synergy scores. All results presented in this section have been computed on the NCI-ALMANAC study restricted to the MCF7 cell line. Combinations are split randomly into training/validation/test (70%/20%/10%). We restrict ourselves to MCF7 for consistency with prospective *in vitro* experiments.

##### Benchmarking on out-of-distribution tasks

In order to understand the out-of-distribution abilities of RECOVER as well as several other models, we evaluate a series of models on six different tasks, described in Figure 3A and S2A. Validation and test metrics are reported in Figure S2C. Test performance can further be visualized in Figures 3B and 3C, as well as Figure S2B.

For this evaluation, the hyperparameters of RECOVER have been optimized (on the validation set of the default task) within the following set of values. Because it was not tractable to perform a grid search over all possible values at once, hyperparameters have been optimized one at a time in an iterative way. The set of parameters that yielded best performance is highlighted, and used for all following experiments (both *in silico* and *in vitro* experiments).

• Learning rate: [$1\times 10^{-1}$, $1\times 10^{-2}$, $1\times 10^{-3}$, $1\times 10^{-4}$, $1\times 10^{-5}$, $1\times 10^{-6}$]
• Batch size: [16, 32, 64, **128**, 256]
• Weight decay: [1, $1\times 10^{-1}$, $1\times 10^{-2}$, $1\times 10^{-3}$, $1\times 10^{-4}$, $1\times 10^{-5}$, $1\times 10^{-6}$, 0]
• Morgan fingerprint radius: [**2**, 3, 4, 5]
• Morgan fingerprint dimension: [**1024**, 2048]
• Output dimension of the single drug MLP: [16, 32, 64, **128**, 256]
• Dimension(s) of the hidden layer(s) of the single drug MLP: [[512], [256], **[1024]**, [2048], [4096], [1024, 1024], [1024, 1024, 1024], [1024, 512], [1024, 512, 256]]
• Dimension(s) of the hidden layer(s) of the combination MLP: [[32], **[64]**, [128], [256], [64, 16], [64, 32], [64, 64]]

Depending on the task at hand, the model configuration will differ slightly. For all tasks (excluding task (iii.)), the drug feature representations consists of the Morgan fingerprints^65^ concatenated with a *one-hot* encoding specifying the identity of drug. For task (iii.), only the Morgan fingerprint is used. When several cell lines are available, the RECOVER model is conditioned on cell lines using feature-wise linear modulation (FiLM).^53^ The cell line features are either a one-hot encoding of the cell line for tasks (iv.) and (vi.), or some information about mutations and basal level of mRNA gene expression for task (v.).

We will now describe a few baseline models and how their hyperparameters have been optimized. A grid search has been performed to optimize the hyperparameters of the *Gradient Boosting Trees* baseline model. The number of trees was set to 100. The set of parameters that yielded best performance is highlighted.

• Maximum tree depth: [2, 5, 10, **20**]
• Minimum number of samples to split a node: [2, 5, 10, **20**, 50]
• Learning rate: [0.0001, 0.001, 0.01, **0.1**, 1]
• Maximum feature: [all, $\sqrt{total\;number\;of\;feature}$,
$\log_{2}\left[\text{total}\;\text{number}\;\text{of}\;\text{features}\right]$]

Similarly, a grid search has been performed for the *Linear SVM* baseline model.

• Tolerance for stopping criterion: [$1\times 10^{-1}$, $1\times 10^{-2}$, $1\times 10^{-3}$, $1\times 10^{-4}$, $1\times 10^{-6}$]
• Regularization C: [0.0001, 0.001, 0.01, **0.1**, 1., 10]

Moreover, we compare against DeepSynergy,^20^ which is a deep learning based approach. We replicated the original model, more precisely:

• Cell line features and drug features were given as input (for all tasks).
• Normalization: Input features’ mean and standard deviation were set to (0,1), followed by a tanh normalization.
• The architecture from the original paper was used (two hidden layers of dimension 8182 and 4096), with input dropout (p=0.2) and layer dropout (p=0.5).
• The final prediction is the average of two predictions made from $\left(d_{1},d_{2}\right)$ and $\left(d_{2},d_{1}\right)$ where $d_{1}$ and $d_{2}$ are the two drugs in the combination.

Finally, we evaluate two variants of RECOVER. In RECOVER (no invariance), the two drug embeddings (outputs of the single drug MLP) are concatenated and directly fed into the combination MLP, instead of first being fed into the invariance module. In RECOVER (shuffled labels), prior to training, drug features are randomly permuted such that each drug gets represented by the features of another drug. A similar procedure is applied to cell line features when they are used, *c.f.* tasks (iv.) to (vi.).

We will now briefly comment the results of the benchmarking study. RECOVER outperforms baseline models in terms of $R^{2}$ and Spearman correlation metrics on the Default task (i.). RECOVER (shuffled labels) performs well compared to other models on the default task, multi cell line task, cell line transfer task and study transfer task. In these cases, the information contained in drug fingerprints and cell line features only provides a limited gain in performance, thus merely knowing the identity of the drugs is sufficient. This is further confirmed by our gradual randomization study, as well as our feature importance study wherein drug structure information only provided a minimal increase in performance on the default task.

In task (iii.) we note a considerable drop in performance when compared to task (i.) for all models alike, demonstrating that RECOVER will have markedly reduced performance when attempting to predict the synergy of drug combinations in which both drugs have not been observed in earlier experiments. The results pertaining to tasks (iv.) and (v.) demonstrate that leveraging experiments from other cell lines does provide a benefit when compared to the performance from task (i.), although the effect is most significant when the specific drug combination in question has been seen in other cell lines, i.e., task (v.). For completion, we confirm the significant batch effects between the NCI-ALMANAC and the O’Neil 2016 studies render using the same model parameters for both studies impossible — notice task (vi.) performing at the level of randomness.

##### Gradual randomization study

In order to further investigate the impact of drug structure information on performance levels, we have performed gradual randomization experiments, wherein a given percentage of the drugs have their representations randomly permuted. Results are shown in Figure S2D. We observe that such randomization has no effect on the default task. This confirms that knowing the identity of the drugs is sufficient in that case.

For other tasks, we can see that performance quickly drops when the randomization percentage increases, meaning that the information contained in drug fingerprints was critical to the performance of the model.

##### Feature importance study

Through investigation of different drug features, we find a large proportion of the performance of RECOVER can be achieved given the identity of the drugs alone, and that structural information allows for a slight increase in performance. As shown in Figure S2G, the performance of the model is similar whether the one hot encoding of the drug or its Morgan fingerprint is used as input. We notice a slight improvement when using both feature types together. Note that the number of parameters of the model is always the same regardless of the type of feature provided as input. When a feature type is not used, the corresponding part of the drug feature vector is set to zero without changing the underlying dimension.

##### Upper bounds on model performance

We investigate RECOVER performance with regards to Spearman correlation and $R^{2}$. Whilst predictive power appears modest, we are still able to identify highly synergistic drug combinations in simulated SMO experiments, see Figure S4D. Several aspects that may limit predictive power: experimental noise, and nonuniformity of maximum Bliss synergy scores.

In Figure S1B, we note most data points are close to zero, with some examples very far from the mean, i.e., the examples of interest. As an example, let us consider the case of Spearman correlation. Given that the observations are noisy, the observed rank among synergies might be corrupted compared to the true ordering — especially in the region close to zero where the density of examples is very high.

The non-uniformity of synergy scores leads to some difficulties in evaluating fairly the performance of RECOVER. For example, the positive tail of the distribution, which is the region of interest, represent a very small percentage of the total number of examples and thus have a little effect on the value of the aggregated statistic.

In order to get a better understanding of the performance of our model, we compare the reported aggregated statistics to an upper bound which takes into account the presence of noise in the observations in addition to the distribution of synergy scores.

We first evaluate the level of noise by considering all replicates from the NCI-ALMANAC study. Two examples are considered replicates when the same pair of drugs has been tested on the same cell line. We found 1960 triplets $\left(d_{1},d_{2},m\right)$ that had been tested several times. For each triplet, we computed the standard deviation of the maximum Bliss score across the replicates. We refer to this as the level of noise for a given triplet. We then computed the average level of noise $\bar{\eta }$ across all triplets.

We then estimate the upper bounds on performance. Given an average level of noise η and the distribution of synergy scores in NCI-ALMANAC, we simulated a noisy acquisition process as follows: the synergies from NCI-ALMANAC were considered as the *true* synergies, and noisy observations were obtained by corrupting the *true* synergies with some Gaussian noise $N\left(0,\eta^{2}\right)$. We then considered a *perfect* regression model which fits the noisy observations exactly, and evaluated its performance on the *true* synergies. Upper bounds are defined by the performance of this *perfect* regression model.

Upper bounds have been computed for $R^{2}$ and Spearman correlation using various levels of noise and are reported in Figure S2F. We see that the noisy acquisition process alone leads to significant limitations in the performance that can be reached. While there is still room for improvement, the performance of RECOVER is reasonably close to the hypothetical maximum. For example, RECOVER achieves 0.47 Spearman correlation, while the highest achievable Spearman correlation is estimated to be 0.64.

##### Performance on the tail of the distribution

We now show that RECOVER achieves good performance on the positive tail of the distribution of synergies, which is necessary to successfully identify highly synergistic combinations within an SMO setting.

Given a model trained on NCI-ALMANAC, we query the top *k* combinations with highest predicted synergy within the test set, and compute the percentage of combinations which are truly synergistic (arbitrarily defined by a maximum Bliss synergy score of above 30) within queried examples. We refer to *k* as the size of the query. In Figure S2E, we report the percentage of synergistic combinations as a function of the size of the query. The percentage of synergistic combinations is superior to the proportion in the whole test set, meaning that the model performs far beyond the level of randomness. For instance, with a query size of 30, we observe a ∼ 5-fold enrichment in synergistic combinations. However, this enrichment decreases with the size of the query.

##### Drug similarity, model uncertainty and error

To help build intuition for the relationship between drug similarity, model uncertainty and model accuracy, we provide some additional results using RECOVER trained on combinations from the ALMANAC database (data pertaining to the MCF7 cell line).

In Figure S4B, we report both model uncertainty (lower left triangle) and mean square error (upper right triangle). Uncertainty is estimated using an ensemble of 20 models. The ordering of the drugs is based on a hierarchical clustering wherein distances between drugs are derived from their Tanimoto similarities. Therefore, the ordering of lines and columns is based on drug structures. Clustering is performed separately for drugs contained within the training/validation set and drugs contained within the test set.

We observe that there is some local consistency in the values of model uncertainty, suggesting that the model is confident in some regions of chemical space, and less confident in other regions of the chemical space. Moreover, we observe columns and rows of high uncertainty, meaning that the model is less confident about specific drugs (regardless of the other drug found in the combination). On average, uncertainty is lowest on the training-validation set, and highest on combinations where neither of the drugs have been seen, *c.f.* marginal distributions of log-uncertainties in Figure S4C (top). Moreover, large model errors seem to preferentially occur in regions of high uncertainty – as shown in Figure S4C. Most importantly, large errors never occur when model is confident (low uncertainty), *c.f.* absence of points in the upper left part of Figure S4C. Furthermore, logged model errors and logged uncertainties are weakly positively correlated (0.22 on the *Training+Validation* split, 0.29 on the *One unseen drug* split, and 0.14 on the *Two unseen drugs* split).

The modeling of structure-activity relationships (SAR) is an extremely difficult task when considering emergent complex phenotypes (e.g., cell death) due to the presence of cliff effects, i.e., there are superficially similar molecules with similar properties, and others with divergent properties. We note that this is such a challenging problem for machine learning that it undoubtedly deserves standalone research without the complicating factor of studying pairs of drugs.

#### SMO development and evaluation (*in silico*)

We benchmarked the SMO pipelines, whereby the model is shown a fraction of the full dataset and can choose sample points to unblind. Our *in silico* experiments try to mirror as closely as possible the setting of the *in vitro* experiments. Therefore, unless specified otherwise, experiments are restricted to the MCF7 cell line and 30 combinations were acquired at a time using the same model as the one used to generate recommendations for the *in vitro* experiments. Uncertainty is estimated using a deep ensemble of size 5, unless stated otherwise.

##### Alternative uncertainty estimator

As deep ensembles are only one method for uncertainty quantification, we tested another approach: directly estimating the uncertainty in the style of DEUP.^55^ Here, two models are initialized: the first one, denoted as the *mean predictor*, predicts the expected synergy $\hat{\mu }$ and is trained with Mean Square Error (MSE). The second model, denoted as the *uncertainty predictor*, outputs an estimate of the standard deviation of the predictive distribution $\hat{\sigma }$ and is trained to minimize the following negative log likelihood criterion:

(Equation 4) $$NLL=\frac{\log\left({\hat{\sigma }}^{2}\right)}{2}+\frac{{\left(y-\hat{\mu }\right)}^{2}}{2{\hat{\sigma }}^{2}}\text{,}$$

where *y* is the ground truth variable of interest. This criterion allows us to get an estimator $\hat{\sigma }$ of the standard deviation of the *predictive* distribution for each combination: given the expected synergy $\hat{\mu }$ predicted by the mean predictor, and the actual observation *y*, we wish to find $\hat{\sigma }$ that maximizes the probability of *y* assuming a predictive distribution of the form $N\left(\hat{\mu },\hat{\sigma }\right)$.

(Equation 5) $$\log\;p\left(y|\hat{\mu },\hat{\sigma }\right)=\log\left[\frac{1}{\sqrt{2\pi {\hat{\sigma }}^{2}}}e^{-\frac{1}{2}{\left(\frac{y-\hat{\mu }}{\hat{\sigma }}\right)}^{2}}\right]$$

(Equation 6) $$=-\frac{1}{2}\log\left(2\pi \right)-\frac{1}{2}\log\left({\hat{\sigma }}^{2}\right)-\frac{1}{2}\frac{{\left(y-\hat{\mu }\right)}^{2}}{{\hat{\sigma }}^{2}}$$

Therefore, maximizing $\log\;p\left(y|\hat{\mu },\hat{\sigma }\right)$ w.r.t. $\hat{\sigma }$ is equivalent to minimizing the NLL criterion presented in Equation 4. Note that only the uncertainty predictor is trained using this criterion. The mean predictor is trained using Mean Square Error (MSE) as we found experimentally that it was more stable. When fixing $\hat{\sigma }=1$, NLL corresponds to the MSE criterion.

##### Efficient exploration of combination space

In order to simulate real-world interactions with the wet lab, we start with a set of 30 randomly chosen drug pairs that the pipeline is initially trained on, while the rest of the data is hidden from the pipeline. Hence, these combinations are always part of the visible set. We now perform an *iteration*: we split the visible set into a training and validation set (80/20), train the model using early stopping and then acquire 30 additional drug pairs from the hidden set. The entire procedure is repeated again with the new training set of 60 drug pairs (i.e., 30+30), and then with 90, and so on, until no more drug pairs are left to acquire from the hidden set. A range of methods can be used to generate recommendations for new drug pairs, typically as specified by an *acquisition function*.^25^

We compare acquisition functions by the rate at which they unblind the set of top 1% of synergistic combinations in the NCI-ALMANAC dataset within each synthetic experimental round (or *iteration*). As shown in Figure S4D (left), Greedy acquisition and Upper Confidence Bound (UCB) acquisition, two model-based strategies, perform on par with each other and outperform the random strategy by a large margin. Both approaches discover 80% of the top 1% of synergistic drug pairs in approximately 15 iterations whereas the random strategy discovers less than 20% in the same number of iterations.

Figure S4D (right) presents the average synergy among queried combinations at each iteration of the SMO pipeline. While the average synergy using random strategy is always approximately 10, model-based strategies query batches for which the average synergy can be up to 25. After approximately 15 iterations, the average synergy in the queries starts to decrease, as there are fewer highly synergistic combinations left to query.

##### Performance of uncertainty driven strategies

When trying to mirror as closely as possible the setting of the *in vitro* experiments, we did not notice any significant difference in performance between the two Model-based acquisition functions Greedy and UCB, as shown in Figures S4D (left). However, depending on the model set up, we can demonstrate that taking uncertainty into account to guide experiments can increase the performance of the pipeline over a naive Greedy acquisition strategy.

In the following, uncertainty was directly estimated using a DEUP-style *uncertainty predictor*, and the task is the prediction of the average Bliss synergy score (instead of maximum Bliss), which corresponds to the average over the dose-response matrix of the concentration specific Bliss scores. In the following, 5 combinations were acquired at a time, instead of 30.

As shown in Figure S4F, UCB can outperform Greedy acquisition, demonstrating the value of taking uncertainty into account in the exploration strategy.

##### Transfer learning: O’Neil 2016 to NCI-ALMANAC

An important aspect of the SMO pipeline is its ability to leverage publicly available data in order to improve performance in a new experimental setting. To this end, we analyzed the impact of pretraining the model on the O’Neil database before simulating SMO experiments on a subset of the NCI-ALMANAC database.

While the out-of-distribution analysis presented in Figure S2C showed that RECOVER does not generalize well to new experimental settings *without adaptation*, these experiments demonstrate, quite remarkably, that some latent knowledge can still be transferred from one experimental setting to another, resulting in increased performance in the latter setting.

As shown in Figure S4E, the model pretrained on O’Neil outperforms the other model, initialized randomly. The model was first pretrained on the O’Neil study, thereafter, we simulated the SMO process on the subset of NCI-ALMANAC consisting of drug pairs for which at least one of the drugs was included in the O’Neil study. We compared against a model that had not been pretrained. All models are conditioned on cell line, and two different uncertainty estimation methods were tested.

In these experiments, we restricted ourselves to cell lines which were covered in both the O’Neil and NCI-ALMANAC studies. As the MCF7 cell line is not included in the O’Neil study, we could not restrict ourselves to MCF7 as usual. Instead, we performed SMO on all overlapping cell lines, resulting in a space of possible queries which is bigger than in other experiments where only MCF7 was used. This explains why the rate of discovery is slower in this case.

### Quantification and statistical analysis

In order to evaluate the significance of the enrichment in highly synergistic combinations within the queries of RECOVER during the prospective evaluation, we performed t tests and a Kolmogorov-Smirnov test. We noted that the mean of the max Bliss synergy scores significantly increases between the first and the third round (t test, p<0.05); this trend further continues by the fifth round (t test, $p<10^{-5}$). Moreover, the distribution starts developing a heavier tail toward high max Bliss synergy scores. This emergent heavy tail already appears significant when comparing the distribution in the first SMO Search round to the background distribution of synergy scores in NCI-ALMANAC (Kolmogorov-Smirnov test, p<0.025).

In Data S1, we report the synergy levels in matrix format and projected on the combination dose-response surface, according to three different synergy models: HSA, Bliss and Loewe. Statistical significance (one sample t test) is computed and reported elementwise (∗ $p<5\times 10^{-2}$, ∗∗ $p<10^{-3}$, ∗∗∗ $p<10^{-4}$; the number of replicates (N) is shown on the top left corner of the matrix display). Note that the colormap only accounts for statistically significant values ($p<5\times 10^{-2}$).

## Data Availability

• All datasets used in this work have been pre-processed, normalized and deposited in a centralized data repository RESERVOIR and are publicly available as of the date of publication. The repository unifies data around relevant molecules and their interactions. Pre-processing and normalizing scripts are provided for traceability, and a Python API has been made available to facilitate access. Access link is also listed in the Key resources table. • All original code has been deposited in a Github python repository (https://github.com/RECOVERcoalition/Recover) and is publicly available as of the date of publication. Our pipeline can be run using custom configuration files. The repository also contains all configurations and visualization scripts used to generate our figures. DOI is also listed in the Key resources table. • Any additional information required to reanalyze the data reported in this work paper is available from the lead contact upon request.

## Supplemental information

Document S1. Figures S1–S4, Data S1, and Table S1

Table S2. Viability assay results for all rounds, related to Figure 4

Document S2. Article plus supplemental information
