# Supplementary material for: RECOVER identifies synergistic drug combinations in vitro through sequential model optimization
Source: Cell Rep Methods. 2023 Oct 4;3(10):100599. doi: 10.1016/j.crmeth.2023.100599 (PMC10626197; doi:10.1016/j.crmeth.2023.100599)
Supplement: Document S1. Figures S1–S4, Data S1, and Table S1 [file mmc1.pdf]

Cell Reports Methods, Volume *3*

Supplemental information

RECOVER identifies synergistic drug combinations *in vitro* through sequential model optimization

Paul Bertin, Jarrid Rector-Brooks, Deepak Sharma, Thomas Gaudelet, Andrew Anighoro, Torsten Gross, Francisco Martínez-Peña, Eileen L. Tang, M.S. Suraj, Cristian Regep, Jeremy B.R. Hayter, Maksym Korablyov, Nicholas Valiante, Almer van der Sloot, Mike Tyers, Charles E.S. Roberts, Michael M. Bronstein, Luke L. Lairson, Jake P. Taylor-King, and Yoshua Bengio

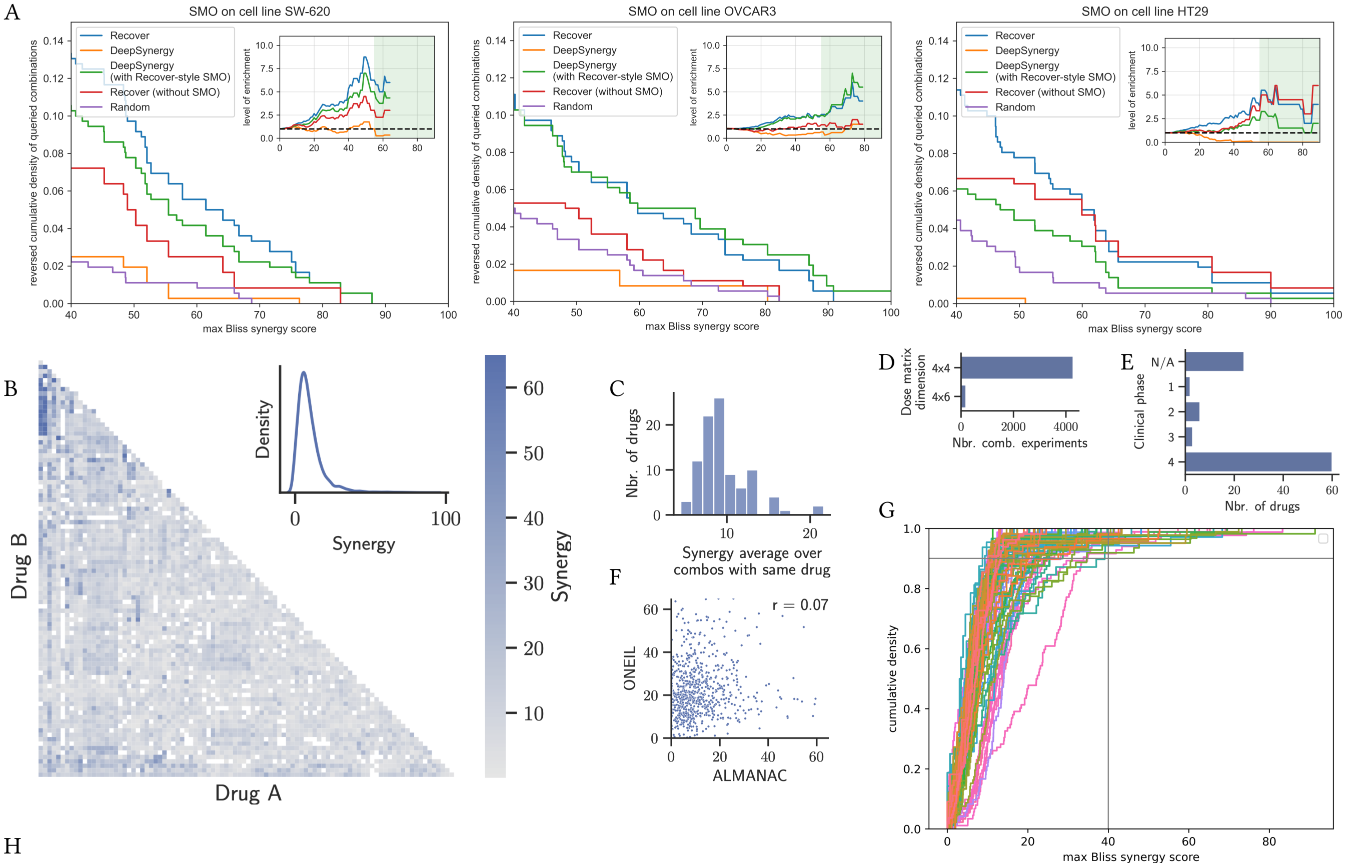

H

Method name	Generalization to new drugs	Generalization to new combos of seen drugs	Prospectively validated	#combos in validation	#cell lines in validation	Stat. significance evaluated	Comment
RECOVER	✓	✓	✓	150	1	✓	Multiple rounds of experimental validation.
ComboFM[5]	✗	✓	✓	16	4	✓	Prospective experiments consist of 16 combos spread across 4 cell lines
ComboNet[4]	✗	✓	✓	30	1	✗	–
DECREASE[3]	✗	✗	✗	–	–	–	Retrospective validation on public and in house datasets (model predictions were not used to select *in vitro* experiments).
IDACombo[6]	✗	✓	✗	–	–	–	Retrospective validation from clinical trials.
DeepSynergy[8]	✗	✓	✗	–	–	–	–
DrugComboRanker[2]	✗	✓	✗	–	–	–	–

Figure S1: Simulations suggest RECOVER can enrich for highly synergistic combinations given a limited budget, related to Figure 2. **(A.)** Reversed cumulative density of queried combinations, following different querying strategies, on three different cell lines. (inset) level of enrichment. We do not provide results for the MCF7 cell line (used in our prospective experiments later on) as this cell line is not part of the O'Neil study, on which models are pretrained. **(B.)** Model performance was evaluated on synergy data from the NCI-ALMANAC viability screen.[1] After quality control, synergy scores could be computed for 4271 unique drug pairs. Synergy scores for each drug-pair on the MCF7 cell line. White squares indicate missing or removed (low-quality) dose-response matrices. **(C.)** Histogram of average synergy scores for each drug computed across all drug pairs recorded. **(D.)** Distribution of dose-response matrix dimensions. **(E.)** Distribution of clinical phases of the considered 95 distinct drugs. **(F.)** Scatter plot across 783 (drug-pair, cell line) tuples found in both NCI-ALMANAC and O'Neil 2016.[7] **(G.)** Cumulative density across all combinations involving a specific drug, for all drugs found in the NCI-ALMANAC study. Analysis restricted to the MCF7 cell line. Gray lines indicate $x = 40$ and $y = 0.9$. **(H.)** Comparison of RECOVER with published works on drug combinations.

A

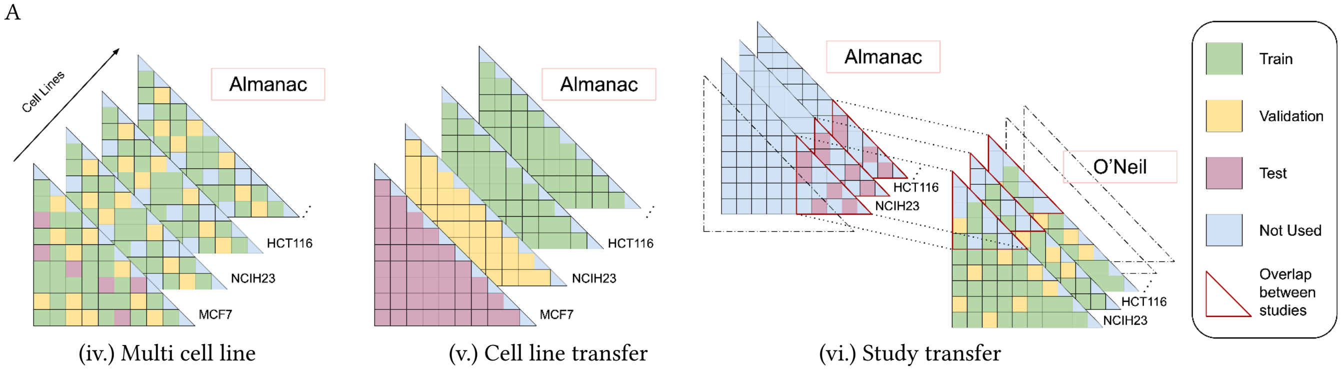

B

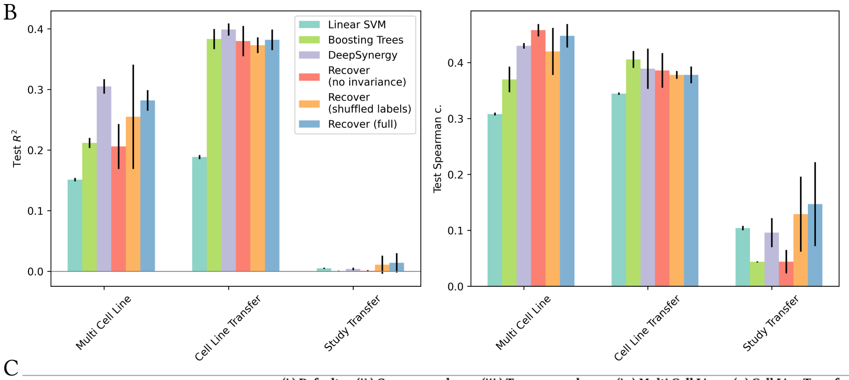

E

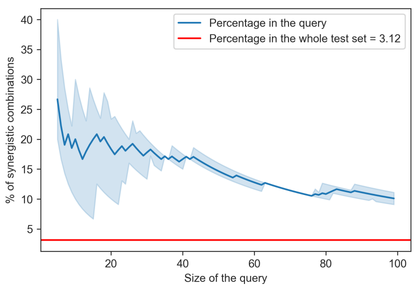

C

			(i.) Default	(ii.) One unseen drug	(iii.) Two unseen drugs	(iv.) Multi Cell Line	(v.) Cell Line Transfer	(vi.) Study Transfer
Linear SVM	R^2	**Valid.**	0.237 ± 0.005	0.221 ± 0.040	0.228 ± 0.055	0.157 ± 0.051	0.142 ± 0.020	0.153 ± 0.013
		**Test**	0.171 ± 0.006	0.167 ± 0.005	0.026 ± 0.008	0.151 ± 0.003	0.189 ± 0.003	0.005 ± 0.001
	Spearman c.	**Valid.**	0.493 ± 0.014	0.440 ± 0.024	0.455 ± 0.045	0.371 ± 0.011	0.373 ± 0.007	0.421 ± 0.009
		**Test**	0.453 ± 0.004	0.333 ± 0.002	0.106 ± 0.015	0.308 ± 0.003	0.344 ± 0.002	0.104 ± 0.004
Gradient Boosting Trees	R^2	**Valid.**	0.276 ± 0.022	0.320 ± 0.084	0.273 ± 0.065	0.294 ± 0.056	0.310 ± 0.014	0.311 ± 0.006
		**Test**	0.172 ± 0.002	0.184 ± 0.011	0.024 ± 0.002	0.212 ± 0.008	0.383 ± 0.017	0.001 ± 0.001
	Spearman c.	**Valid.**	0.484 ± 0.033	0.475 ± 0.042	0.430 ± 0.022	0.474 ± 0.010	0.507 ± 0.008	0.580 ± 0.010
		**Test**	0.459 ± 0.001	**0.362 ± 0.002**	0.137 ± 0.015	0.370 ± 0.023	0.406 ± 0.015	0.044 ± 0.001
DeepSynergy	R^2	**Valid.**	0.329 ± 0.034	0.283 ± 0.079	0.356 ± 0.111	0.393 ± 0.015	0.366 ± 0.011	0.378 ± 0.021
		**Test**	0.144 ± 0.025	0.170 ± 0.012	**0.059 ± 0.003**	**0.305 ± 0.012**	**0.399 ± 0.010**	0.004 ± 0.002
	Spearman c.	**Valid.**	0.453 ± 0.011	0.424 ± 0.056	0.401 ± 0.032	0.520 ± 0.006	0.350 ± 0.045	0.640 ± 0.009
		**Test**	0.454 ± 0.008	0.302 ± 0.018	**0.176 ± 0.003**	0.430 ± 0.005	**0.389 ± 0.036**	0.096 ± 0.026
RECOVER (no invariance)	R^2	**Valid.**	0.332 ± 0.074	0.275 ± 0.064	0.330 ± 0.123	0.361 ± 0.057	0.301 ± 0.017	0.358 ± 0.032
		**Test**	0.208 ± 0.001	**0.195 ± 0.008**	0.043 ± 0.005	0.206 ± 0.037	0.380 ± 0.025	0.001 ± 0.001
	Spearman c.	**Valid.**	0.457 ± 0.036	0.471 ± 0.051	0.455 ± 0.073	0.504 ± 0.007	0.294 ± 0.023	0.625 ± 0.008
		**Test**	0.437 ± 0.003	0.329 ± 0.006	0.145 ± 0.023	**0.458 ± 0.011**	**0.386 ± 0.031**	0.044 ± 0.021
RECOVER (shuffled labels)	R^2	**Valid.**	0.325 ± 0.058	0.298 ± 0.086	0.384 ± 0.161	0.391 ± 0.030	0.271 ± 0.028	0.320 ± 0.020
		**Test**	0.237 ± 0.014	0.086 ± 0.015	0.003 ± 0.003	0.255 ± 0.086	0.373 ± 0.013	0.011 ± 0.015
	Spearman c.	**Valid.**	0.460 ± 0.009	0.466 ± 0.053	0.439 ± 0.070	0.510 ± 0.019	0.268 ± 0.063	0.598 ± 0.032
		**Test**	0.449 ± 0.016	0.228 ± 0.016	−0.050 ± 0.04	0.420 ± 0.042	0.378 ± 0.007	0.129 ± 0.067
RECOVER(full)	R^2	**Valid.**	0.343 ± 0.053	0.275 ± 0.067	0.401 ± 0.147	0.387 ± 0.032	0.278 ± 0.027	0.304 ± 0.021
		**Test**	**0.242 ± 0.006**	**0.196 ± 0.008**	0.038 ± 0.002	0.282 ± 0.017	0.382 ± 0.017	**0.014 ± 0.016**
	Spearman c.	**Valid.**	0.474 ± 0.021	0.433 ± 0.023	0.459 ± 0.069	0.518 ± 0.021	0.299 ± 0.047	0.589 ± 0.032
		**Test**	**0.466 ± 0.007**	0.326 ± 0.025	0.157 ± 0.012	**0.448 ± 0.021**	**0.378 ± 0.015**	**0.147 ± 0.075**

F

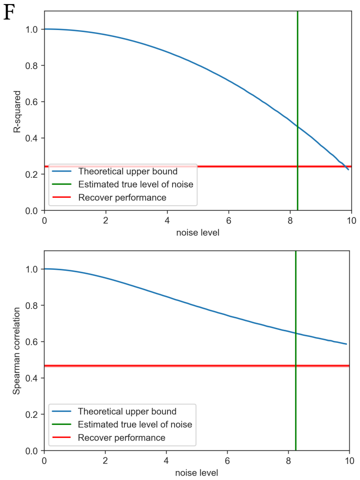

D

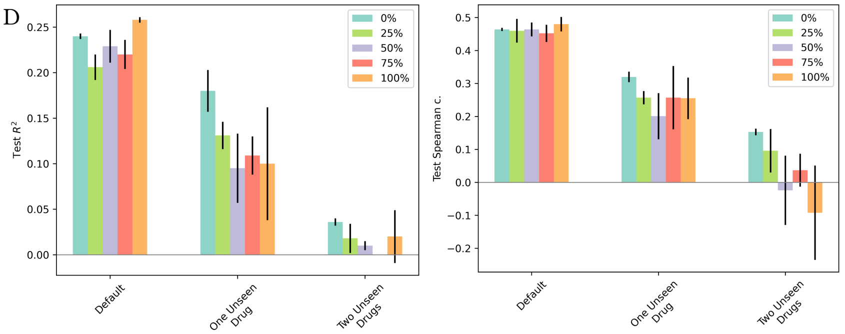

G

	R^2	Spearman corr.
RECOVER (fingerprint + one hot)	**0.242 ± 0.006**	**0.466 ± 0.007**
RECOVER (fingerprint)	0.232 ± 0.007	0.458 ± 0.010
RECOVER (one hot)	0.230 ± 0.029	0.449 ± 0.017

Figure S2: Retrospective testing demonstrates the ability of RECOVER to generalize when at least one of the drugs has been seen during training, but fails to generalize across studies, related to Figure 3. **(A.)** Overview of the different tasks on which RECOVER has been evaluated within the *personalised framework*. **(iv.)** Multi Cell Line. All cell lines are used for training and validation, but the test set is restricted to the MCF7 cell line. If a combination is part of the test set, it is never used in the training and validation sets, regardless of cell line. Training/validation split is consistent across cell lines. Proportions (70%/20%/10%) **(v.)** Cell Line Transfer. The test set consists of all examples corresponding to the MCF7 cell line. Other cell lines are randomly assigned to training/validation (80%/20%). A given combination can appear in both training and test sets **(vi.)** Study Transfer. The O'Neil 2016 study is used to generate the training and validation sets. All overlapping cell lines are used. The test set is generated using NCI-ALMANAC and restricted to combinations for which both drugs also appear in the O'Neil study. If a combination is part of the test set, it is excluded from training and validation sets, regardless of cell line. **(B.) and (C.)** Performance of RECOVER and other models for the three different tasks. **(D.)** Effect of gradual randomization of drug identities on model performance for the different tasks. **(E.)** Percentage of highly synergistic combinations (synergy score > 30) in queried combinations using RECOVER as a function of the size of the query. **(F.)** Comparison of the performance of RECOVER (default task) with upper bounds that take into account the level of noise in the data, and the non uniform distribution of synergies. The upper bound, shown in blue, is a function of the level of noise. The standard deviation for the upper bound is lower than 10^{-3} for both statistics. The estimated level of noise in our dataset is shown in green. The performance of RECOVER as well as its standard error are shown in red. **(G.)** Feature importance study. For all panels, standard deviation computed over 3 seeds.

A

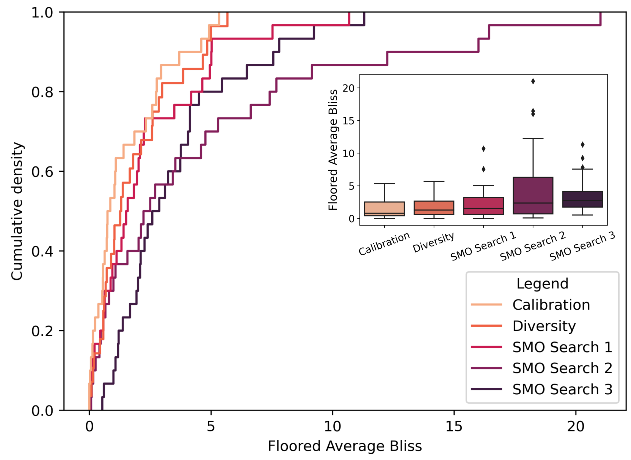

Floored average Bliss: average of Bliss scores floored at zero (to ignore antagonistic effects) across all concentration pairs: $\frac{1}{|C_a||C_b|}\sum_{c_a,c_b\in C_a,C_b}\max(S(c_a,c_b),0)$.

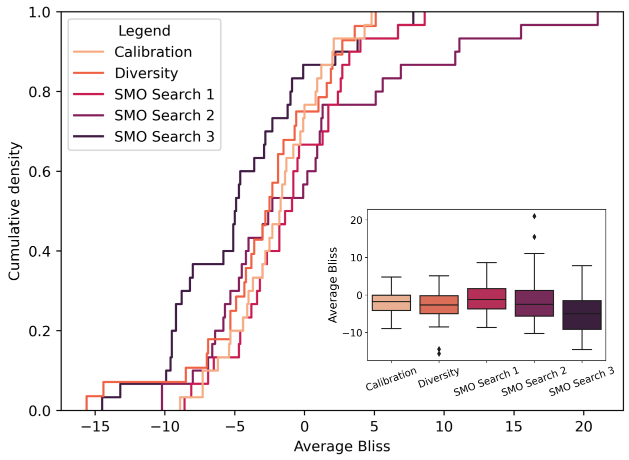

Average Bliss: $\frac{1}{|C_a||C_b|}\sum_{c_a,c_b\in C_a,C_b} S(c_a,c_b)$

B

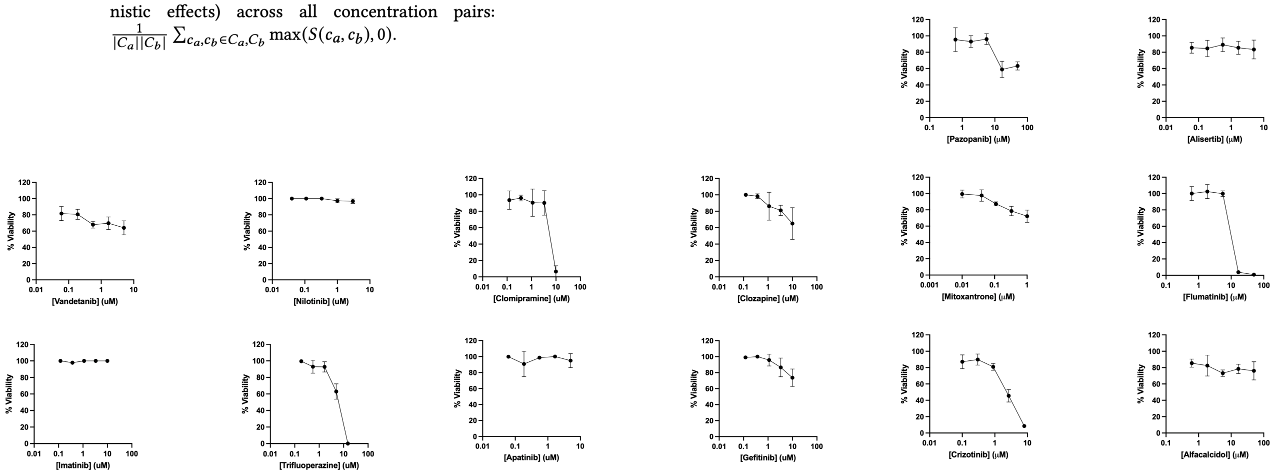

C

Compound A	Compound B	Loewe (avg)	Bliss (avg)	HSA (avg)	Loewe (max)	Bliss (max)	HSA (max)
Crizotinib	Alfacalcidol	81.85	81.85	81.85	99.99	99.99	99.99
Gefitinib	Alfacalcidol	58.90	44.28	63.82	61.77	60.26	61.79
Gefitinib	Clomipramine	29.76	31.68	35.07	67.53	62.32	69.70
Imatinib	Alfacalcidol	45.87	45.87	45.87	57.70	57.70	57.70
Imatinib	Apatinib	22.62	23.90	24.11	62.52	62.52	62.52
Imatinib	Clomipramine	16.60	16.60	16.60	64.04	64.04	64.04
Imatinib	Clozapine	30.43	31.56	34.46	59.51	54.98	66.34
Imatinib	Trifluoperazine	41.66	41.66	41.66	62.96	62.96	62.96
Mitoxantrone	Flumatinib	14.68	20.14	23.99	45.76	50.38	53.98
Nilotinib	Clomipramine	23.96	23.96	23.96	49.58	49.58	49.58
Pazopanib	Alisertib	11.64	12.09	14.29	51.77	47.84	51.77
Pazopanib	Flumatinib	11.85	13.29	13.53	38.97	39.17	39.23
Pazopanib	Trifluoperazine	22.62	21.51	22.80	48.61	44.83	48.61
Vandetanib	Alfacalcidol	54.43	30.72	58.01	62.30	52.75	62.30

Figure S3: *In vitro* evaluation demonstrates the significant enrichment for highly synergistic combinations through prospective use of RECOVER, related to Figure 4. **(A.)** Cumulative density plots of aggregated Bliss synergy score for each experimental round, for two different aggregation strategies; (insets) box plot representations. **(B.)** Single agent dose response viability curves before normalization. **(C.)** Synergies according to Combenefit for 14 highly synergistic combinations queried prospectively.

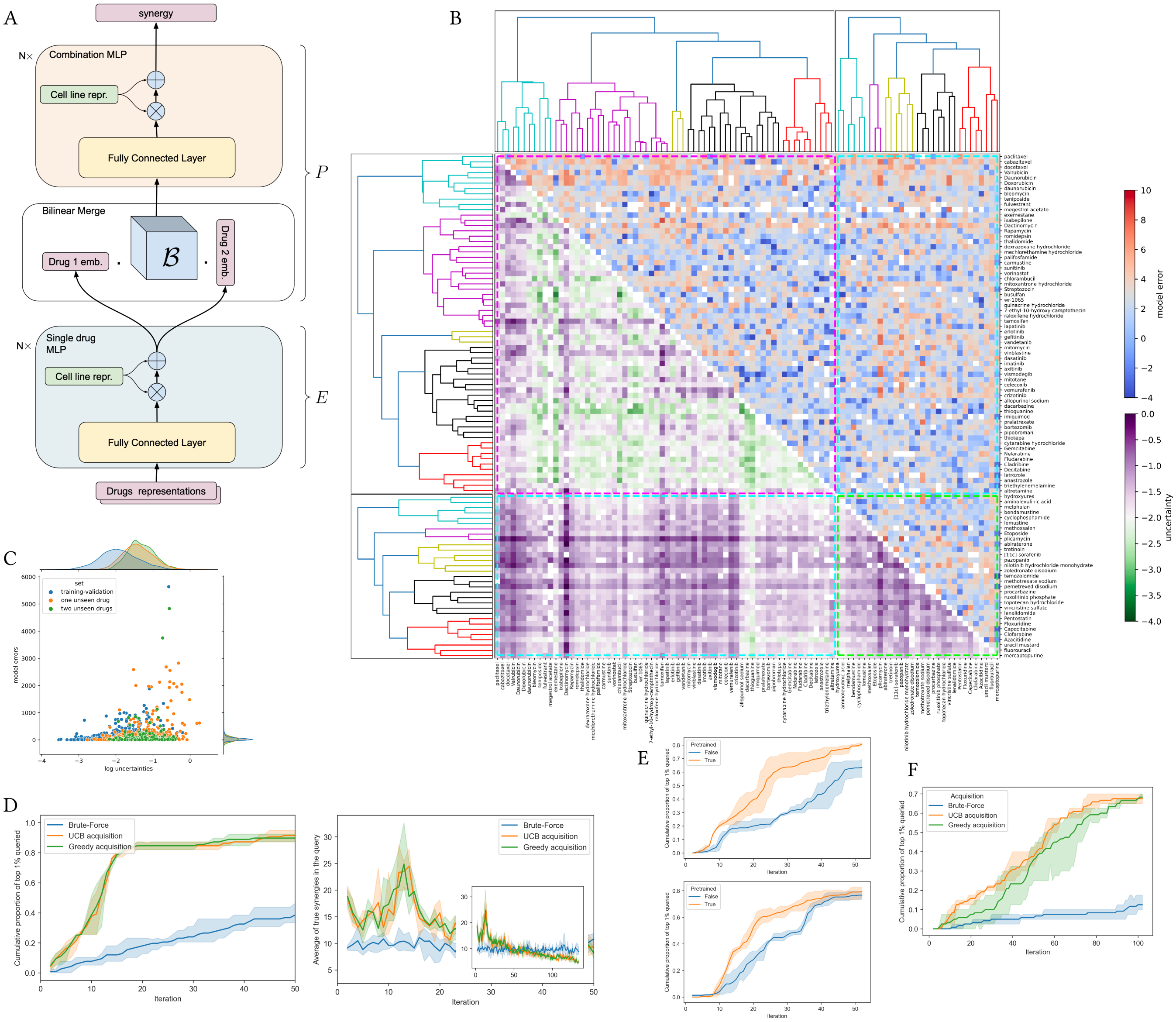

Figure S4: Overview of the RECOVER model and *in silico evaluation*, related to STAR Methods. **(A.)** Drug representations are fed into the Single drug module which is composed of an MLP which can be conditioned on cell line. Given two drug embeddings, the synergy is predicted using the Combination module, composed of a bilinear operation followed by an MLP. The Combination MLP can be conditioned on cell line as well. **(B.)** Uncertainty of RECOVER (lower left triangle) and mean square error (upper right triangle). The magenta square corresponds to the training-validation set. The part of the matrix outside of the magenta square corresponds to the test set, which can be subdivided into two parts: combinations where one of the drugs has been seen during training (cyan rectangles), and combinations where none of the drugs have been seen during training (light green square). The ordering of the drugs is based on a hierarchical clustering wherein distances between drugs are derived from their Tanimoto similarities. The color map is on a logarithmic scale, both for model error and for model uncertainty. White entries correspond to combinations absent from the dataset. **(C.)** Errors made by the RECOVER model as a function of its log-uncertainty. Color corresponds to the dataset split to which the combination belongs. **(D.)** Comparison of acquisition functions through *in silico* SMO experiments: *(left)* Cumulative proportion of the top 1% synergistic combinations that have been rediscovered by RECOVER, *(right)* Average of the true synergies of the drug pairs that have just been queried at each iteration of the SMO pipeline, *(inset)* Zoomed out view. Uncertainty estimated via deep ensembles. **(E.)** Effect of pretraining on the rate of discovery of highly synergistic combinations via a UCB acquisition function: *(top)* Deep Ensemble, *(bottom)* Direct Uncertainty Estimation. Evaluation performed on the subset of NCI-ALMANAC consisting of drug pairs for which at least one of the drugs is included in O'Neil. All overlapping cell lines were included. **(F.)** Comparison of acquisition functions through in silico SMO experiments. Prediction of the average Bliss synergy score. 5 combinations acquired at a time. Uncertainty estimated using direct uncertainty estimation. For all panels, standard deviation computed over 3 seeds.

Data S1: Detailed results for top synergistic combinations (page 1/7), related to Figure 4.

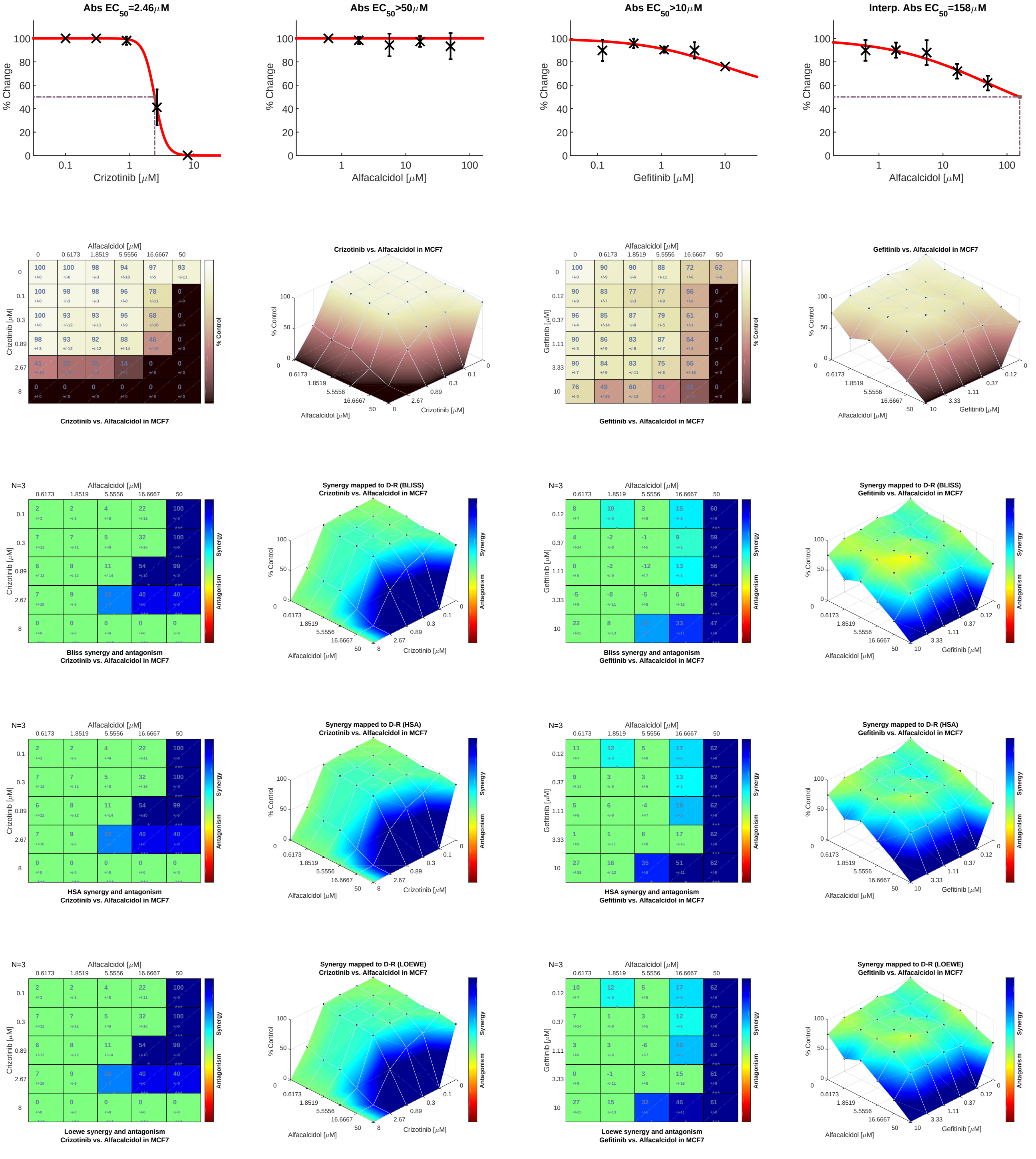

Crizotinib vs. Alfacalcidol

Gefitinib vs. Alfacalcidol

Data S1: Detailed results for top synergistic combinations (page 2/7), related to Figure 4.

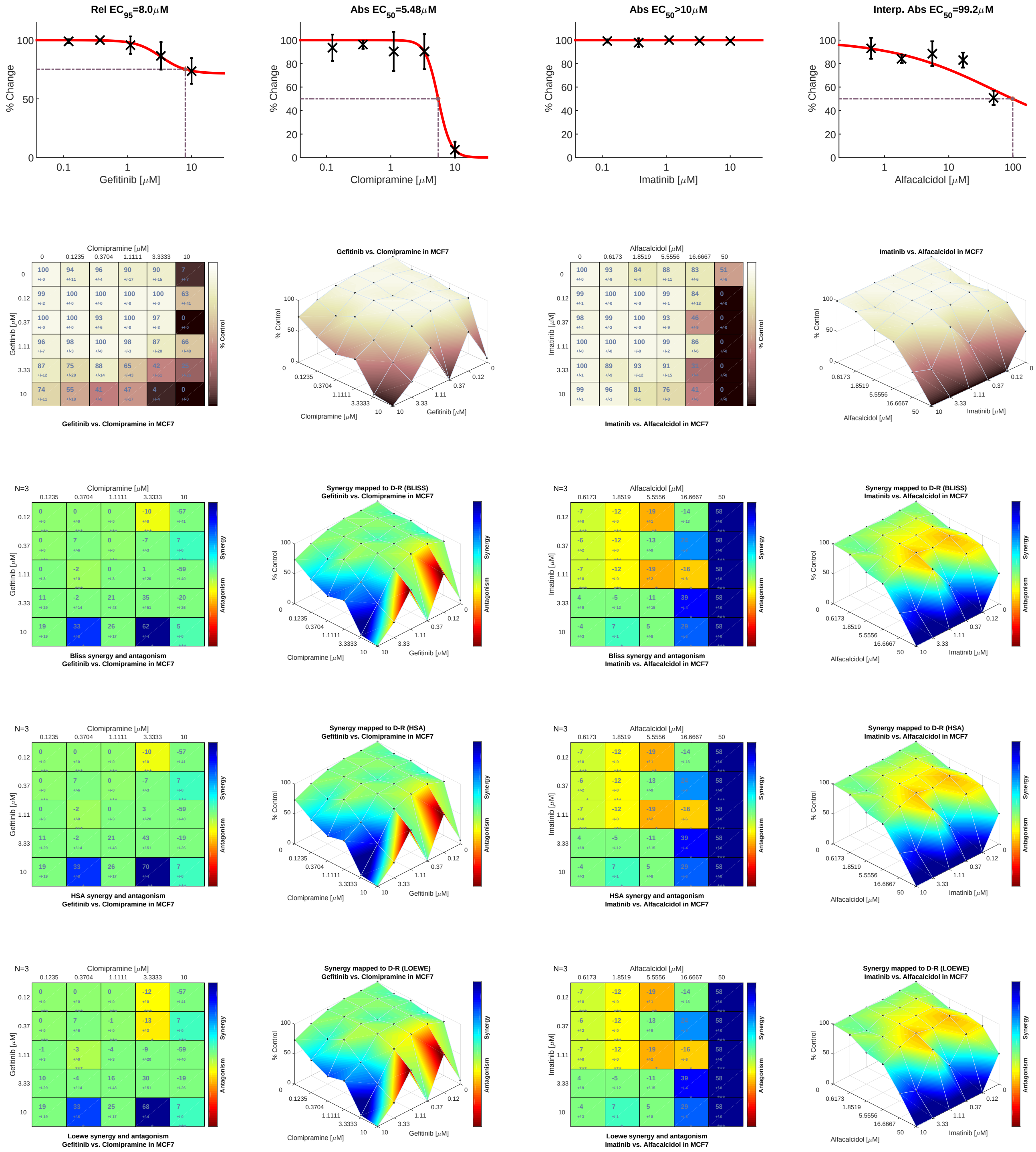

Gefitinib vs. Clomipramine

Imatinib vs. Alfacalcidol

Data S1: Detailed results for top synergistic combinations (page 3/7), related to Figure 4.

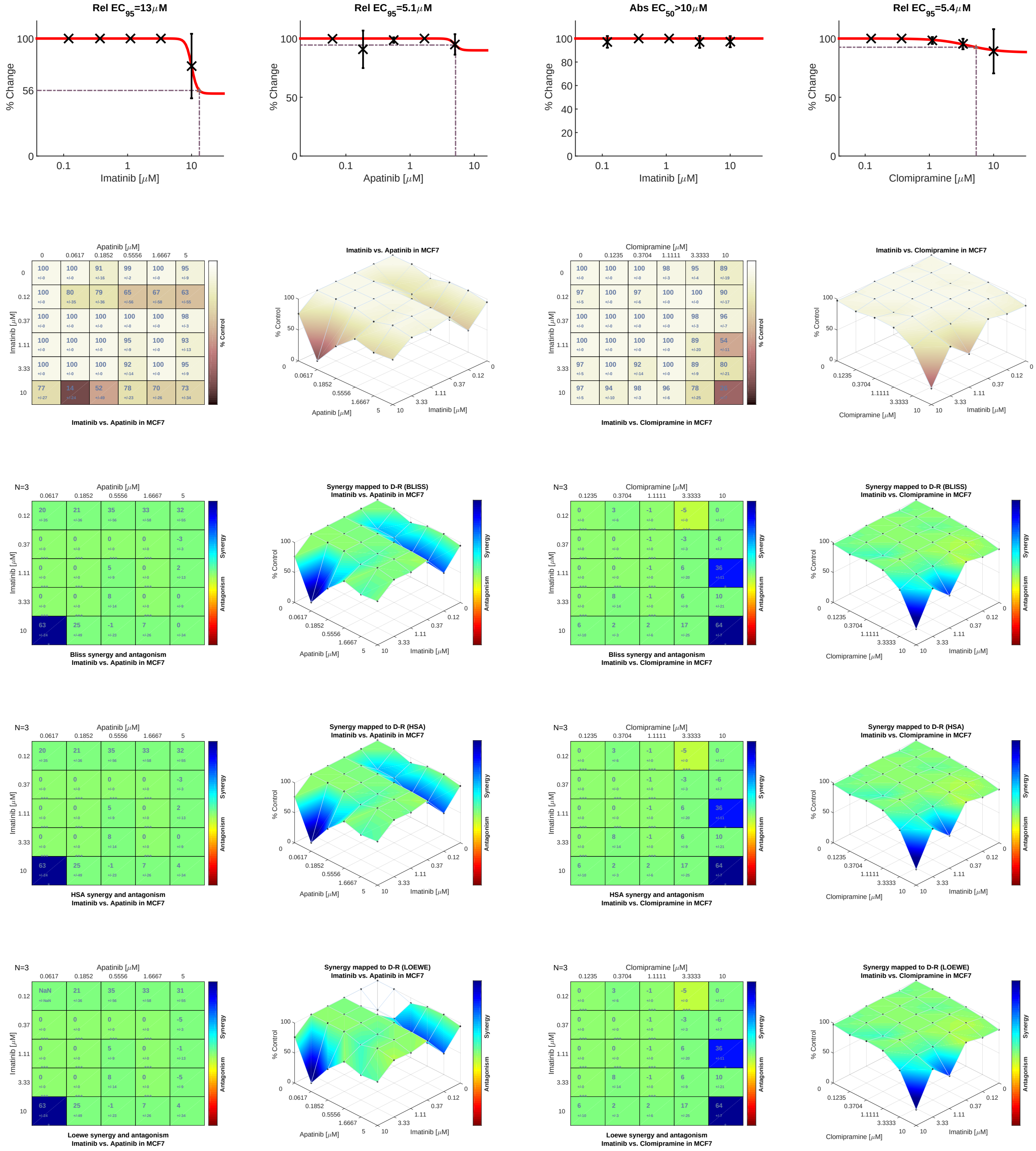

Imatinib vs. Apatinib

Imatinib vs. Clomipramine

Data S1: Detailed results for top synergistic combinations (page 4/7), related to Figure 4.

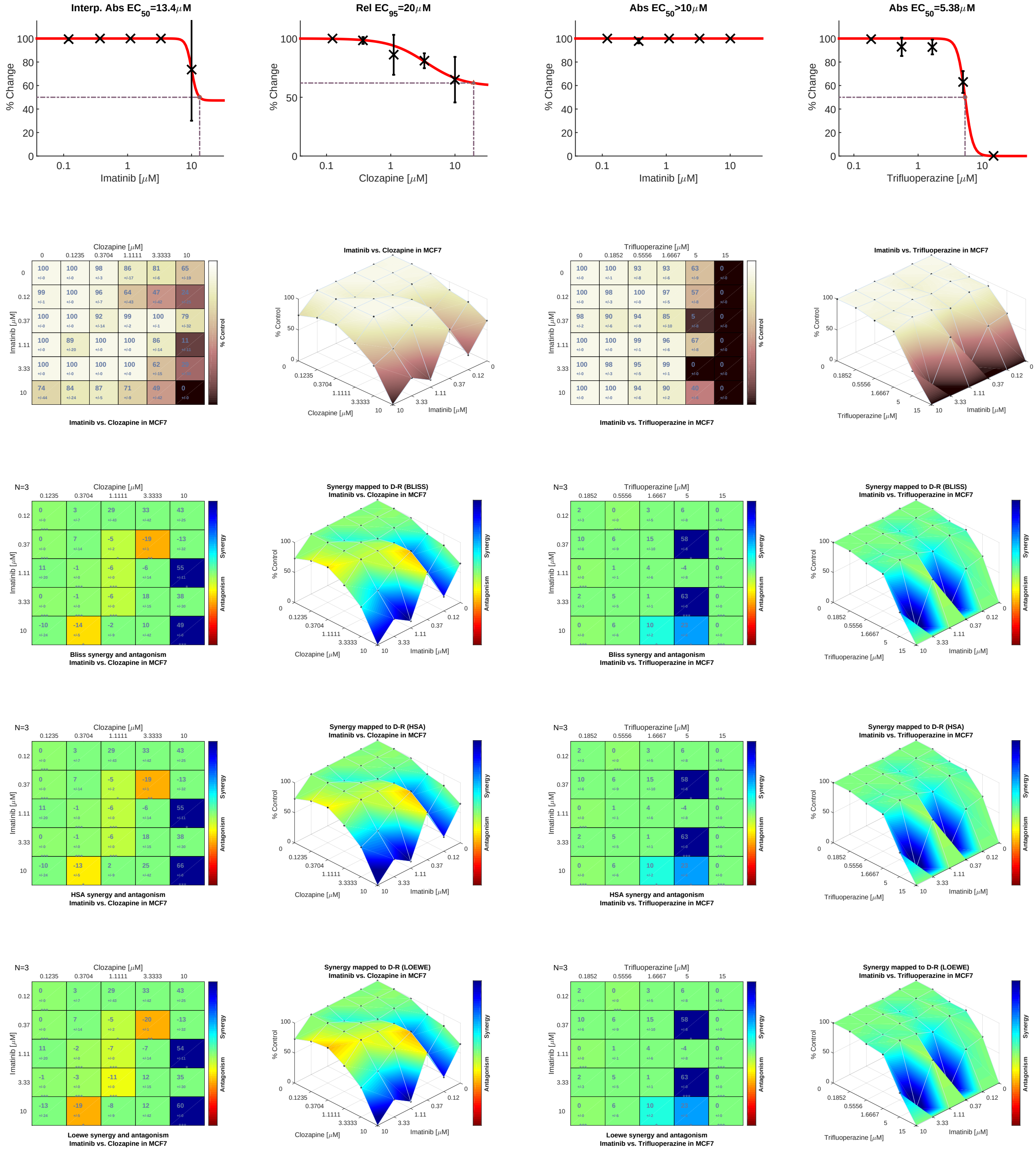

Imatinib vs. Clozapine

Imatinib vs. Trifluoperazine

Data S1: Detailed results for top synergistic combinations (page 5/7), related to Figure 4.

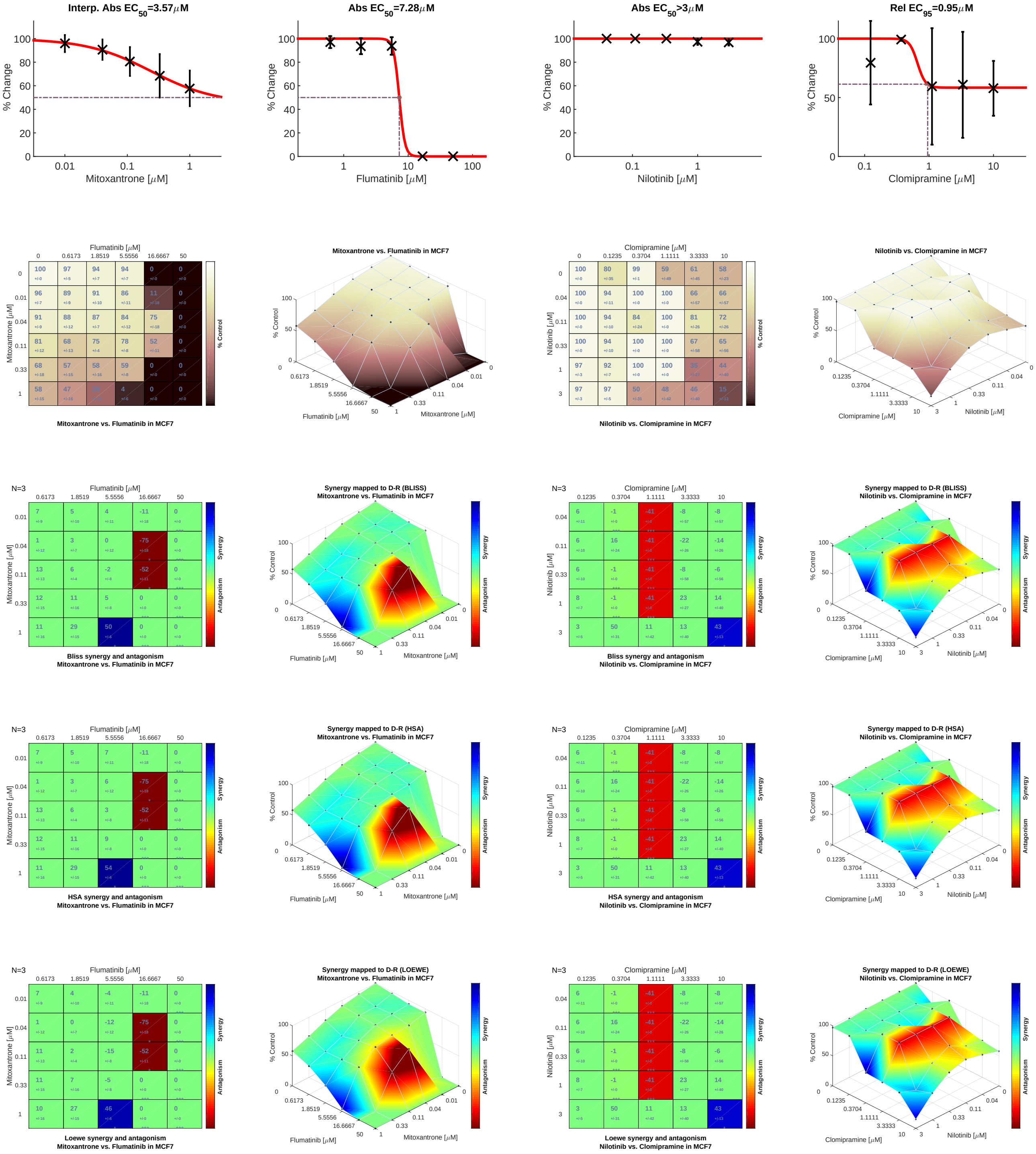

Mitoxantrone vs. Flumatinib

Nilotinib vs. Clomipramine

Data S1: Detailed results for top synergistic combinations (page 6/7), related to Figure 4.

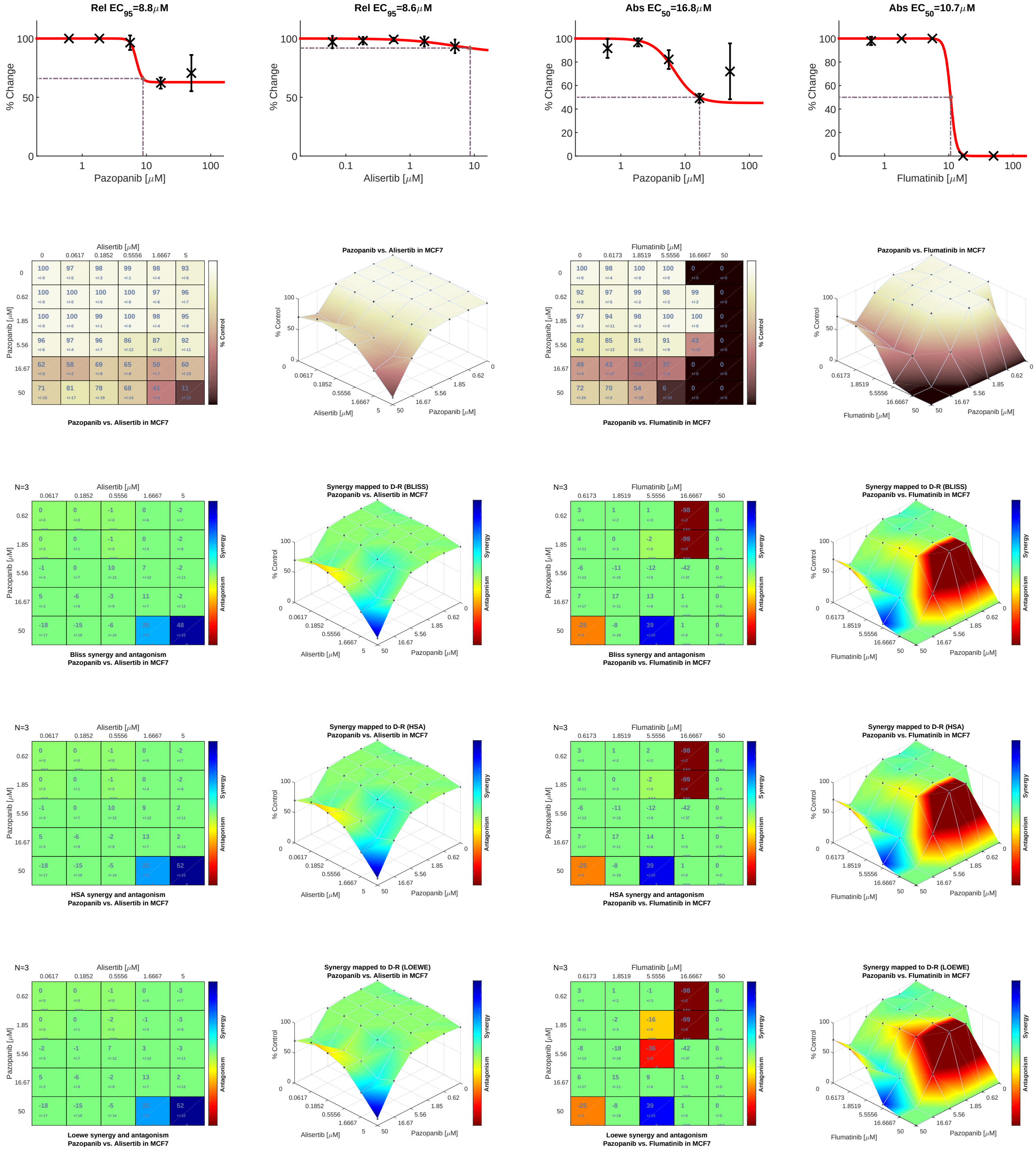

Pazopanib vs. Alisertib

Pazopanib vs. Flumatinib

Data S1: Detailed results for top synergistic combinations (page 7/7), related to Figure 4.

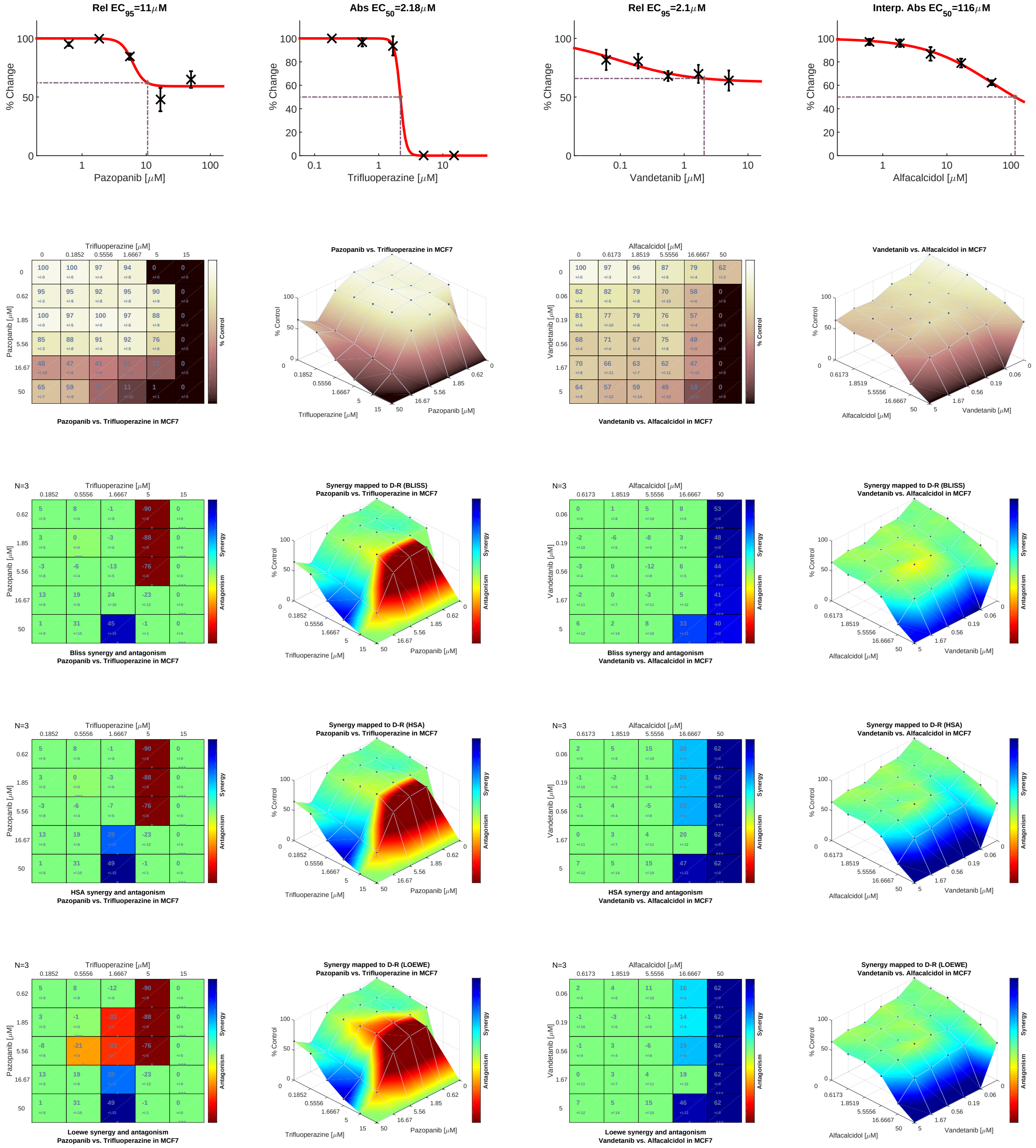

Pazopanib vs. Trifluoperazine

Vandetanib vs. Alfacalcidol

Table S1: Aggregated synergy scores, related to Figure 4.

Drug 1 name	Drug 2 name	Round	max Bliss	average Bliss	Floored average Bliss
5-Fluorouracil	Foretinib	Calibration	0.1304325084	0.01101740201	0.02442144056
Abiraterone	Entinostat	Calibration	0.2115434669	0.02090925759	0.04960187652
Allopurinol	Terazosin (hydrochloride dihydrate)	Calibration	0.06941114319	-0.0173562375	0.007061821924
Anastrozole	Gedatolisib (PF-05212384; PKI-587)	Calibration	0.09174160745	0.02244498344	0.02696429089
Axitinib	Progesterone	Calibration	0.1012446273	-0.07383087463	0.005576645254
Cabazitaxel	Flumatinib (mesylate)	Calibration	0	-0.02753543938	0
Carmustine	Clomipramine (hydrochloride)	Calibration	0.1575197918	0.04774210248	0.05325820935
Crizotinib	Clofibrate	Calibration	0.00977498684	-0.03634407566	0.001370364371
Daunorubicin	Loperamide (hydrochloride)	Calibration	0.09377301063	-0.01271469392	0.01438641442
Docetaxel	13-cis-Retinoic acid	Calibration	0.2043830815	0.009436799177	0.03759445138
Hydroxyurea	Orantinib	Calibration	0.06764636797	-0.02450557865	0.007498975508
Imiquimod	Theophylline	Calibration	0.03143476727	-0.07127652179	0.001257390691
Lapatinib	Prednisolone	Calibration	0.1224498136	-0.01444860284	0.01081265351
Lenalidomide	Itacitinib	Calibration	0.03951592677	-0.00815799215	0.005519442765
Letrozole	Dopamine HCl	Calibration	0.05634072859	-0.003409441946	0.005575382038
Megestrol	Rapamycin	Calibration	0.04788923667	-0.04094138293	0.007393298746
Methotrexate	5-Azacytidine	Calibration	0.07969955995	-0.03789306478	0.008720139143
Mitoxantrone	Poziotinib	Calibration	0.1436465344	-0.00003520243936	0.02976219175
Paclitaxel	BMS-582949 (hydrochloride)	Calibration	0.1910215425	0.001802883819	0.02898244451
Pazopanib	Dacomitinib	Calibration	0.07332752397	-0.02918889215	0.00616765495
Pralatrexate	Cinacalcet (hydrochloride)	Calibration	0.1045193689	-0.0154711988	0.01103302551
Raloxifene	Estradiol	Calibration	0.0005138362533	-0.08519720394	0.00002055345013
Sorafenib	Sumatriptan (succinate)	Calibration	0.01201447449	-0.0507863276	0.0007522213569
Tamoxifen	Naproxen	Calibration	0.1059263476	-0.01771159088	0.01045587378
Topotecan	Betaxolol (hydrochloride)	Calibration	0.007868423763	-0.06100215454	0.0003147369505
Vandetanib	Ramipril	Calibration	0.1263467553	-0.0237003127	0.01849475348
Vinblastine	Toremifene (citrate)	Calibration	0.2622059597	0.01273802119	0.02814259151
Vincristine	Bazedoxifene (acetate)	Calibration	0.1895627224	0.04273457941	0.04627377709
Zoledronic	Loratadine	Calibration	0.03728378293	-0.04393742813	0.003696762644
ixabepilone	Spebrutinib	Calibration	0.037580886	-0.05273488181	0.002363529877
5-Fluorouracil	Valdecoxib	Diversity	0.1076860413	-0.05368429624	0.007041013804
Abiraterone	Ruboxistaurin (LY333531 HCl)	Diversity	0.06565317445	-0.02285983162	0.01025945564
Axitinib	PKI-166	Diversity	0.06249663491	-0.05312855408	0.006064620107
Cabazitaxel	Alisertib	Diversity	0.02375247221	-0.1405623062	0.0009500988882
Cabazitaxel	Gedatolisib (PF-05212384; PKI-587)	Diversity	0.1096408788	0.01290513372	0.02740039558
Cabazitaxel	Itacitinib	Diversity	0.1502283989	-0.01944198022	0.0253886142
Cabazitaxel	Terazosin (hydrochloride dihydrate)	Diversity	0.03549820988	-0.06754378112	0.001914019335
Crizotinib	Tosedostat	Diversity	0.1576916171	0.01997117808	0.03886155101
Docetaxel	JNJ-38877605	Diversity	0.1705975349	-0.02725193406	0.0180717791
Erlotinib	Alfacalcidol	Diversity	0.4060613787	0.05117413977	0.05673355706
Gefitinib	Hydrochlorothiazide	Diversity	0.05164335683	-0.02528168261	0.00573637266
Imatinib	Atracurium (besylate)	Diversity	0.2723489484	-0.03521332963	0.02874594311
Megestrol	Trifluoperazine 2HCl	Diversity	0.3022667117	0.03649186127	0.04699516564
Methotrexate	Methocarbamol	Diversity	0.07351226459	-0.005387268254	0.0131389337
Mitoxantrone	Dutasteride	Diversity	0.1625364026	0.01575356815	0.04844613013
Paclitaxel	lenvatinib Mesylate	Diversity	0.06313485539	-0.06429313528	0.004538745097
Pazopanib	GSK2636771	Diversity	0.06950403106	-0.0824500547	0.01015701269
Raloxifene	Agomelatine	Diversity	0.02283796861	-0.04665674081	0.001533131619
Ruxolitinib	Betamethasone dipropionate	Diversity	0.1098345273	-0.01490711603	0.006935240011
Tamoxifen	Prostaglandin E1	Diversity	0.07014474233	-0.0370568728	0.005924406638
Vandetanib	Epinephrine HCl	Diversity	0.1735033849	0.01939573608	0.03035921279

Vemurafenib	Apatinib	Diversity	0.2412556069	-0.03037126291	0.02140230633
Vinblastine	Clozapine	Diversity	0.2390638498	0.02598368032	0.0492124743
Vincristine	Sotrastaurin	Diversity	0.06811477935	-0.004514570368	0.01711865514
Vismodegib	PD0325901	Diversity	-0.01683018041	-0.1522635663	0
ixabepilone	Progesterone	Diversity	0.1275727384	-0.01824960784	0.01380556683
ixabepilone	Ribociclib	Diversity	0.1511064341	-0.0387655626	0.01135415817
ixabepilone	Terazosin (hydrochloride dihydrate)	Diversity	0.1550944088	-0.04161340101	0.01278251825
Abiraterone	Alfacalcidol	SMO Search 1	0.1370420178	-0.00949679762	0.01849716485
Cabazitaxel	BMS-582949 (hydrochloride)	SMO Search 1	0.01904555518	-0.0320450315	0.0007618222073
Cabazitaxel	Dacomitinib	SMO Search 1	0.2424523262	-0.02626680009	0.01590414862
Cabazitaxel	Foretinib	SMO Search 1	0.07695050725	0.01677409824	0.02290833832
Cabazitaxel	Ribociclib	SMO Search 1	0.1536683698	-0.03019335749	0.01405436984
Cabazitaxel	Spebrutinib	SMO Search 1	0.1018262245	-0.03775749424	0.006200040407
Erlotinib	Alfacalcidol	SMO Search 1	0.4341098176	0.08579924643	0.1067873304
Erlotinib	Apatinib	SMO Search 1	0.08744742315	0.01688758116	0.02020528917
Erlotinib	BMS-582949 (hydrochloride)	SMO Search 1	0.1277290745	0.02621165842	0.03492759341
Erlotinib	Clomipramine (hydrochloride)	SMO Search 1	0.120483758	0.01350517206	0.02123562257
Erlotinib	Terazosin (hydrochloride dihydrate)	SMO Search 1	0.03748313064	-0.01772371278	0.006240501658
Erlotinib	Trifluoperazine 2HCl	SMO Search 1	0	-0.06049498629	0
Megestrol	Alfacalcidol	SMO Search 1	0.296058699	0.02856090891	0.05035613002
Mitoxantrone	BMS-582949 (hydrochloride)	SMO Search 1	0.03874578191	-0.07657224737	0.001549831276
Mitoxantrone	Clonidine	SMO Search 1	0.02927401906	-0.06700273863	0.002069426466
Mitoxantrone	Flumatinib (mesylate)	SMO Search 1	0.5067806542	-0.006984276731	0.05033063359
Pazopanib	Alfacalcidol	SMO Search 1	0.230890944	0.05132437459	0.05836033442
Pazopanib	Alisertib	SMO Search 1	0.5541456112	0.0244202427	0.04684563638
Vemurafenib	Alfacalcidol	SMO Search 1	0.1709271883	-0.004019118754	0.01825232541
Vemurafenib	Trifluoperazine 2HCl	SMO Search 1	0.1224567772	-0.03406217012	0.01107586707
Vincristine	BMS-582949 (hydrochloride)	SMO Search 1	-0.01308099719	-0.08395533806	0
Vincristine	Flumatinib (mesylate)	SMO Search 1	0.08271546758	-0.04359100856	0.004597391033
Vincristine	Loperamide (hydrochloride)	SMO Search 1	0.1347842546	-0.01781409451	0.01334582268
Vincristine	Toremifene (citrate)	SMO Search 1	0.2126421755	0.03234436767	0.04186208837
Vincristine	Trifluoperazine 2HCl	SMO Search 1	0.304397299	0.06740959068	0.0752859265
ixabepilone	Alfacalcidol	SMO Search 1	0.1483191721	-0.008913185355	0.02235825102
ixabepilone	Alisertib	SMO Search 1	0.07967362346	-0.04647781204	0.01508939282
ixabepilone	Apatinib	SMO Search 1	0.06246539057	-0.01389998136	0.008667747411
ixabepilone	Dacomitinib	SMO Search 1	0.06204994357	-0.04382371767	0.005543011443
ixabepilone	Gedatolisib (PF-05212384; PKI-587)	SMO Search 1	0.04533062751	-0.001281414548	0.01042498528
Cabazitaxel	Alfacalcidol	SMO Search 2	0.2244634842	0.01312907389	0.05282963906
Cabazitaxel	Sotrastaurin	SMO Search 2	0.03407917561	-0.04046082032	0.002513449673
Crizotinib	Alfacalcidol	SMO Search 2	0.9333212112	0.2110150215	0.2115473702
Erlotinib	Alfacalcidol	SMO Search 2	0.3827106222	0.06958084308	0.09136831284
Gefitinib	Alfacalcidol	SMO Search 2	0.5940499177	0.1568425158	0.1656293587
Imatinib	Alfacalcidol	SMO Search 2	0.5131557335	0.1115117863	0.160064849
Imatinib	Alisertib	SMO Search 2	0.08936474141	-0.05579242721	0.0114412282
Imatinib	Flumatinib (mesylate)	SMO Search 2	0.01867801369	-0.05802525557	0.0010129612
Imatinib	Trifluoperazine 2HCl	SMO Search 2	0.6298601129	0.05112731673	0.06636482891
Lapatinib	Flumatinib (mesylate)	SMO Search 2	0.1255842166	0.0112622542	0.02025230131
Megestrol	Alisertib	SMO Search 2	0.1883669088	0.01173078952	0.03492351143
Methotrexate	Flumatinib (mesylate)	SMO Search 2	0.3156518429	0.007977516741	0.07687879684
Mitoxantrone	Alisertib	SMO Search 2	0.01290016172	-0.06389710678	0.0009226782337
Mitoxantrone	Bazedoxifene (acetate)	SMO Search 2	0.3145591213	-0.001094216929	0.02474371979
Mitoxantrone	JNJ-38877605	SMO Search 2	0.03905308646	-0.04581174804	0.00485546742
Mitoxantrone	Toremifene (citrate)	SMO Search 2	0.1244317493	0.007915395052	0.02612746216
Mitoxantrone	Trifluoperazine 2HCl	SMO Search 2	0.07562682358	-0.1011526119	0.006899930603

Pazopanib	Clomipramine (hydrochloride)	SMO Search 2	0.3978047001	0.05626885796	0.07422184574
Pazopanib	Flumatinib (mesylate)	SMO Search 2	0.6578092306	-0.06814770002	0.04801390213
Pazopanib	Prednisolone	SMO Search 2	0.07517579448	-0.02551057984	0.01549228828
Pazopanib	Tosedostat	SMO Search 2	0.1660147628	-0.02916839785	0.02246044312
Pazopanib	Trifluoperazine 2HCl	SMO Search 2	0.4986788491	-0.08007341028	0.04584383021
Vandetanib	Alfacalcidol	SMO Search 2	0.5086136465	0.1084934853	0.1227007674
Vemurafenib	Alisertib	SMO Search 2	0.1626997637	-0.02056685201	0.03500797293
Vemurafenib	Ribociclib	SMO Search 2	0.2138334403	0.002404419526	0.02118711562
Vinblastine	Flumatinib (mesylate)	SMO Search 2	0.123113372	-0.04197787578	0.009907255405
Vincristine	Alisertib	SMO Search 2	0.05331091677	-0.09581875388	0.005693754852
Vincristine	Clozapine	SMO Search 2	0.1265016037	-0.04530589912	0.00803449926
Vincristine	JNJ-38877605	SMO Search 2	0.07120488509	-0.0523464671	0.005710691335
Vincristine	Spebrutinib	SMO Search 2	0.03984171228	-0.06385581116	0.001593668491
Crizotinib	Apatinib	SMO Search 3	0.07768785541	-0.02877137271	0.01216208155
Crizotinib	Bazedoxifene (acetate)	SMO Search 3	0.1732910936	-0.04885085362	0.01678905259
Crizotinib	Clomipramine (hydrochloride)	SMO Search 3	0.1972862679	-0.08634747845	0.02052872967
Crizotinib	Entinostat	SMO Search 3	0.1570674581	-0.07250719987	0.03763195393
Crizotinib	Ribociclib	SMO Search 3	0.1212561769	-0.01049252966	0.01493449418
Gefitinib	Apatinib	SMO Search 3	0.2093379395	-0.03802145153	0.02582210813
Gefitinib	Bazedoxifene (acetate)	SMO Search 3	0.5276515754	-0.02351273821	0.07817004094
Gefitinib	Clomipramine (hydrochloride)	SMO Search 3	0.6269807662	-0.01000289959	0.07967017663
Gefitinib	Entinostat	SMO Search 3	0.1500460036	-0.09505632047	0.01990300997
Gefitinib	Ribociclib	SMO Search 3	0.3854460872	-0.08195721231	0.0470495024
Imatinib	Apatinib	SMO Search 3	0.6241480257	0.07787290716	0.09198397763
Imatinib	Bazedoxifene (acetate)	SMO Search 3	0.2764843593	-0.09264992146	0.03056303362
Imatinib	Clomipramine (hydrochloride)	SMO Search 3	0.6123107266	0.038662778	0.05463635832
Imatinib	Clozapine	SMO Search 3	0.5363960351	0.07096097232	0.1144111533
Imatinib	Entinostat	SMO Search 3	0.3618720269	-0.05651149704	0.03755003218
Methotrexate	Bazedoxifene (acetate)	SMO Search 3	0.2136498039	-0.08770157992	0.02691272025
Methotrexate	Clozapine	SMO Search 3	0.1898201002	-0.009381414347	0.04018665117
Methotrexate	Dacomitinib	SMO Search 3	0.05595483315	-0.04389605846	0.008053339418
Methotrexate	Entinostat	SMO Search 3	0.08242208559	-0.069167129	0.01432773359
Methotrexate	Itacitinib	SMO Search 3	0.2230787852	0.02048452271	0.04058360031
Nilotinib	Apatinib	SMO Search 3	0.1275266037	-0.08194267747	0.01255186846
Nilotinib	Bazedoxifene (acetate)	SMO Search 3	0.2905089432	-0.008546340088	0.02491556588
Nilotinib	Clomipramine (hydrochloride)	SMO Search 3	0.4650303858	-0.05708119886	0.06545916859
Nilotinib	Entinostat	SMO Search 3	0.209701004	-0.04677225641	0.03583810275
Nilotinib	Ribociclib	SMO Search 3	0.0551705757	-0.1446847165	0.005309835602
Vandetanib	Apatinib	SMO Search 3	0.198043113	-0.04003616473	0.02308893434
Vandetanib	Clomipramine (hydrochloride)	SMO Search 3	0.2709781743	-0.1122000547	0.04398342231
Vandetanib	Clozapine	SMO Search 3	0.2742327451	0.003339949801	0.04483573982
Vandetanib	Ribociclib	SMO Search 3	0.2139974178	-0.09944326373	0.01931882169
Vismodegib	Ribociclib	SMO Search 3	0.2536916482	-0.03436567587	0.02326534242

REFERENCES

[1] S. L. Holbeck, R. Camalier, J. A. Crowell, J. P. Govindharajulu, M. Hollingshead, L. W. Anderson, E. Polley, L. Rubinstein, A. Srivastava, D. Wilsker, et al., The National Cancer Institute ALMANAC: a comprehensive screening resource for the detection of anticancer drug pairs with enhanced therapeutic activity, Cancer Research, 77 (2017), pp. 3564–3576.

[2] L. Huang, F. Li, J. Sheng, X. Xia, J. Ma, M. Zhan, and S. T. Wong, Drugcomboranker: drug combination discovery based on target network analysis, Bioinformatics, 30 (2014), p. i228–i236.

[3] A. Ianevski, A. K. Giri, P. Gautam, A. Kononov, S. Potdar, J. Saarela, K. Wennerberg, and T. Aittokallio, Prediction of drug combination effects with a minimal set of experiments, Nature Machine Intelligence, 1 (2019), p. 568–577.

[4] W. Jin, J. M. Stokes, R. T. Eastman, Z. Itkin, A. V. Zakharov, J. J. Collins, T. S. Jaakkola, and R. Barzilay, Deep learning identifies synergistic drug combinations for treating covid-19, Proceedings of the National Academy of Sciences, 118 (2021).

[5] H. Julkunen, A. Cichonska, P. Gautam, S. Szedmak, J. Douat, T. Pahikkala, T. Aittokallio, and J. Rousu, Leveraging multi-way interactions for systematic prediction of pre-clinical drug combination effects, Nature Communications, 11 (2020), p. 6136.

[6] A. Ling and R. S. Huang, Computationally predicting clinical drug combination efficacy with cancer cell line screens and independent drug action, Nature Communications, 11 (2020), p. 5848.

[7] J. O'Neil, Y. Benita, I. Feldman, M. Chenard, B. Roberts, Y. Liu, J. Li, A. Kral, S. Lejnine, A. Loboda, et al., An unbiased oncology compound screen to identify novel combination strategies, Molecular Cancer Therapeutics, 15 (2016), pp. 1155–1162.

[8] K. Preuer, R. P. Lewis, S. Hochreiter, A. Bender, K. C. Bulusu, and G. Klambauer, Deepsynergy: predicting anti-cancer drug synergy with deep learning, Bioinformatics, 34 (2018), pp. 1538–1546.
